# Dynamic Changes in Jackfruit (*Artocarpus heterophyllus* Lam.) Polyphenols During Maturation and In Vitro Digestion and Their Effects on the Gut Microbiota

**DOI:** 10.3390/antiox15010026

**Published:** 2025-12-23

**Authors:** Liru Ma, Zhen Feng, Chao Zhang, Chunhe Gu, Yihong Bao

**Affiliations:** 1 College of Life Science, Northeast Forestry University, Harbin 150040, China; maliru@nefu.edu.cn; 2 Spice and Beverage Research Institute, Chinese Academy of Tropical Agricultural Sciences, Wanning 571533, China; neau_fengzhen@163.com (Z.F.); zc8477371@126.com (C.Z.); 3 Key Laboratory of Processing Suitability and Quality Control of the Special Tropical Crops, Wanning 571533, China; 4 Key Laboratory of Dairy Science, Ministry of Education, College of Food Science, Northeast Agricultural University, Harbin 150030, China; 5 Key Laboratory of Forest Food Resources Utilization of Heilongjiang Province, Harbin 150040, China

**Keywords:** jackfruit, polyphenols, maturation, in vitro digestion, colonic fermentation, gut microbiota

## Abstract

Jackfruit (*Artocarpus heterophyllus* Lam.) phenolic compounds are chemically diverse. Previous studies have predominantly focused on easily extractable free polyphenols, with limited attention on bound (conjugated) polyphenols. The transformation mechanisms of these polyphenols during maturation, digestion, and colonic fermentation remain unclear, limiting the full utilization of jackfruit resources. This study provides a comprehensive portrait of how jackfruit polyphenols shift in abundance, chemical composition, and bioactivity throughout maturation, in vitro digestion, and colonic fermentation. It also quantifies their selective reshaping of the human gut microbiota and their enhancement of short-chain fatty acid production. The results indicate a significant conversion of bound to free polyphenols during maturation, with distinct behaviors observed across different digestion stages. More importantly, jackfruit polyphenols selectively enrich beneficial gut bacteria, promote short-chain fatty acid production, and positively regulate gut health.

## 1. Introduction

Jackfruit (*Artocarpus heterophyllus* Lam.) is an evergreen species belonging to the Moraceae family and the genus Artocarpus [1]. It is widely cultivated in India, Thailand, Vietnam, Malaysia, and South China [2]. Its distinct flavor is highly accepted by consumers. Both immature and mature fruits have considerable processing value. Mature fruits are typically used to produce canned products, jellies, jams, and candies. Immature fruits are mainly consumed as vegetables in dishes such as curries and salads [3]. Jackfruit contains abundant bioactive compounds, particularly polyphenols [4]. These secondary metabolites are widely distributed in plants and are characterized by multiple phenolic hydroxyl groups attached to aromatic rings. They mainly comprise phenolic acids, flavonoids, and other phenolic compounds [5]. Polyphenols are classified into free and bound forms based on their association with the plant matrix [6]. Bound polyphenols can be further separated into soluble conjugated phenols and insoluble bound phenols based on their solubility.

Polyphenols exhibit multiple bioactivities, including antioxidant, hypoglycemic, hypolipidemic, and hypouricemic effects. They also modulate gut microbiota composition and enhance the production of short-chain fatty acids [7]. Their antioxidant activity is primarily attributed to free radical scavenging and metal ion chelation. Common in vitro assays include the DPPH (2,2-diphenyl-1-picrylhydrazyl) radical scavenging assay, the ABTS (2,2′-azino-bis(3-ethylbenzothiazoline-6-sulfonic acid)) radical cation scavenging assay, and the FRAP (ferric reducing antioxidant power) assay. Regarding hypoglycemic activity, extensive in vitro, animal, and clinical studies have shown that polyphenols can reduce hyperglycemia, improve insulin sensitivity, promote insulin secretion, and mitigate secondary complications of type 2 diabetes [8]. Alpha-glucosidase inhibition is an effective strategy for managing type 2 diabetes, as the enzyme hydrolyzes dietary carbohydrates into glucose, contributing to postprandial hyperglycemia [9]. Polyphenols can also help restore gut microbiota balance in individuals with obesity [10]. In addition, pancreatic lipase—an important metabolic target—hydrolyzes 50–70% of dietary fat into fatty acids and glycerol, which subsequently participate in lipid synthesis. Therefore, inhibition of this enzyme is considered an effective approach for controlling obesity [11]. Polyphenols may also reduce uric acid synthesis by inhibiting xanthine oxidase [1], a major therapeutic target for hyperuricemia [12]. Notably, polyphenols selectively promote the growth of beneficial gut bacteria and modulate the overall microbial composition. Gut microbes play a central role in nutrient digestion, absorption, and secondary metabolism by regulating intestinal barrier function and producing short-chain fatty acids [13].

During fruit ripening, polyphenols undergo extensive transformations caused by hydrolysis mediated by endogenous glycosidases and oxidation catalyzed by phenoloxidases [14]. However, most current studies focus on free polyphenols in fully ripe fruits. Studies on bound polyphenols—including soluble conjugated and insoluble bound forms—and on their interconversion during ripening remain limited. This gap limits the efficient utilization of jackfruit resources. It is noteworthy that bound phenols exhibit substantial bioactivities, particularly in relation to gut health [15]. Moreover, the bioactivities of polyphenols are strongly influenced by their transformations during digestion and colonic fermentation [16]. Cianciosi et al. [17] reported that some microbial metabolites exhibit markedly stronger bioactivities than their precursor compounds. However, their specific transformation pathways remain unclear. Therefore, comprehensive analysis of polyphenol transformations during digestion and colonic fermentation is essential. In vivo experiments are time-consuming and complex, and they are constrained by ethical considerations. Moreover, elucidating metabolic pathways in the digestive tract remains challenging [18]. Consequently, in vitro digestion and colonic fermentation models are widely used to investigate the transformation of bioactive compounds [19].

Based on our previous findings that unripe jackfruit exhibits higher biological activity, it is essential to elucidate how jackfruit polyphenols of different maturities behave during gastrointestinal transit. This study aims to track the structural evolution of jackfruit residues during in vitro digestion and colonic fermentation and to quantify how ripeness influences polyphenol content, composition, antioxidant capacity, and inhibition of α-glucosidase, pancreatic lipase, and xanthine oxidase along the digestive continuum. Furthermore, this study evaluates how these ripeness-dependent transformations modulate the human gut microbiota and short-chain fatty acid production, providing a basis for the targeted utilization of jackfruit at different maturity stages in functional food development.

## 2. Materials and Methods

### 2.1. Plant Material

Jackfruit Thai No. 5 (T5) was harvested in June at the experimental site of the Spice and Beverage Research Institute in Wanning, Hainan Province, China (18°15′ N, 110°13′ E). The fruits underwent post-harvest ripening at 28 ± 1 °C and 70–75% relative humidity (RH), with three ripening stages identified based on phenotypic characteristics (Figure S1): J1 (unripe), J2 (underripe), and J3 (ripe). After the seeds were removed, the jackfruit pulp was lyophilized, powdered, sieved (40 mesh), and stored at −20 °C. All chemical and bioactivity assays (including total phenolics, flavonoids, antioxidant capacity, etc.) were performed using samples from all three maturity stages (J1, J2, and J3), which were processed in parallel to allow direct comparisons across the ripening continuum.

#### Maturity Classification of Jackfruit

To ensure uniform physiological maturity, the fruits were categorized into three maturity stages (J1, J2, J3) based on external color, textural firmness, aroma intensity, and latex exudation. The criteria were as follows:

J1 (unripe): The fruit has a vivid green surface with sharply pointed, closely spaced spines. The flesh is rock-hard and does not yield to finger pressure. The aroma is limited to grassy notes, with no typical jackfruit odor. When cut, a large amount of white, sticky latex exudes. The bulbs are underdeveloped, white, crisp, and starchy, making them suitable only for culinary processing.

J2 (underripe): The skin turns yellow-green to light brown, and the spines are blunted and spaced further apart. The fruit remains firm but shows slight spring-back when pressed, with a faint fruity aroma detectable. Latex flow is markedly reduced. The bulbs are cream to pale yellow, partially developed, and beginning to accumulate soluble sugars, representing the stage for commercial harvest and the onset of ripening.

J3 (ripe): The rind is golden-yellow to brownish yellow, with flattened spines and externally swollen fruit. The flesh is soft and easily indented with light thumb pressure, emitting a strong sweet fragrance. Latex is virtually absent. The bulbs are bright golden, plump, juicy, and soft, with maximal total soluble solids and flavor, making them ideal for fresh consumption.

All fruit was field-graded by trained assessors, strictly adhering to the above descriptors.

### 2.2. Chemical Agent

#### 2.2.1. Reagents and Enzyme

The reagents and enzymes used in this study were obtained from the following sources: 1,1-diphenyl-2-picrylhydrazyl (DPPH), 2,2′-azino-bis(3-ethylbenzothiazoline-6-sulfonic acid) (ABTS), 2,4,6-tripyridyl-s-triazine (TPTZ), porcine pancreatic lipase, disodium phenyl phosphate (pNPP), chromatographic formic acid, and spectroscopic potassium bromide were purchased from Aladdin Biochemical Technology Co., Ltd. (Shanghai, China). Additionally, alpha-amylase (50,000 U/g), pepsin (30,000 U/g), trypsin (2500 U/mg), bile salts (cholic acid content ≥ 60%), antioxidant standard Trolox, α-glucosidase, p-nitrophenyl-α-D-glucopyranoside (PNPG), xanthine oxidase, and xanthine were supplied by Shanghai YuanYe Biotechnology Co., Ltd. (Shanghai, China). Mass spectrometry-grade methanol and acetonitrile were obtained from Sigma-Aldrich (St. Louis, MO, USA). Folin–Ciocalteu reagent was acquired from Solaibao Technology Co., Ltd. (Beijing, China), and ethyl acetate was sourced from Guangdong Xilong Scientific Co., Ltd. (Guangdong, China).

#### 2.2.2. Calibration Standard

Eighteen calibration standards were used for HPLC quantification, including the following phenolic and flavonoid markers: gallic acid, chlorogenic acid, p-hydroxybenzoic acid, caffeic acid, homovanillic acid, 3-hydroxyphenylacetic acid, sinapic acid, ferulic acid, 2-methoxycinnamaldehyde, (+)-catechin, vitexin-2″-O-rhamnoside, hyperoside, (−)-epicatechin gallate, taxifolin, glycitein, quercetin, naringenin, and mulberrin, all of which were purchased from Shanghai YuanYe Biotechnology Co., Ltd. (Shanghai, China).

### 2.3. In Vitro Digestion and Colon Fermentation

#### 2.3.1. In Vitro Digestion

Following the method of Minekus et al. [20], simulated saliva fluid (SSF), simulated gastric fluid (SGF), and simulated intestinal fluid (SIF) were prepared. For SSF preparation, a mixture of 15.1 mL of 0.5 M KCl, 3.7 mL of 0.5 M KH_2_PO_4_, 6.8 mL of 1 M NaHCO_3_, 0.5 mL of 0.15 M MgCl_2_·(H_2_O)_6_, 0.06 mL of 0.5 M (NH_4_)_2_CO_3_, and 373.84 mL of ultrapure water was prepared to a final volume of 400 mL. For SGF preparation, 6.9 mL of 0.5 M KCl, 0.9 mL of 0.5 M KH_2_PO_4_, 12.5 mL of 1 M NaHCO_3_, 11.8 mL of 2 M NaCl, 0.4 mL of 0.15 M MgCl_2_·(H_2_O)_6_, 0.5 mL of 0.5 M (NH_4_)_2_CO_3_, and 367 mL of ultrapure water were mixed to a final volume of 400 mL. For SIF preparation, 6.8 mL of 0.5 M KCl, 0.8 mL of 0.5 M KH_2_PO_4_, 42.5 mL of 1 M NaHCO_3_, 9.6 mL of 2 M NaCl, 1.1 mL of 0.15 M MgCl_2_·(H_2_O)_6_, and 339.2 mL of ultrapure water were mixed to a final volume of 400 mL.

In the oral phase (OP), 200 mg of jackfruit powder was dispersed in 0.5 mL of 200 mM PBS. Subsequently, 3.5 mL of SSF, 0.5 mL of 1500 U/mL α-amylase solution, and 25 μL of 0.3 M CaCl_2_(H_2_O)_2_ were added. After homogenization, the pH was adjusted to 7.0, and the mixture was incubated at 37 °C for 2 min. In the gastric phase (GP), the oral digesta were mixed with 3.75 mL of SGF, 0.8 mL of 25,000 U/mL pepsin solution, and 2.5 μL of 0.3 M CaCl_2_(H_2_O)_2_. After adjusting the pH to 3.0, the mixture was incubated at 37 °C for 2 h. In the intestinal phase (IP), the gastric digesta was mixed with 5.5 mL of SIF, 2.5 mL of 800 U/mL trypsin solution, 1.25 mL of 0.15% bile salt solution, and 20 μL of 0.3 M CaCl_2_(H_2_O)_2_. After adjusting the pH to 7.0, the mixture was incubated at 37 °C for 2 h.

#### 2.3.2. In Vitro Colonic Fermentation

All procedures were conducted in accordance with the Declaration of Helsinki and relevant national regulations and were approved by the Science and Technology Ethics Committee of Northeast Forestry University (Harbin, China, Approval No. 2025114). Written informed consent was obtained from each participant after the nature and potential consequences of the study were fully explained. Fresh fecal samples were collected from five healthy adult volunteers (three males and two females, aged 20–30 years) with no history of gastrointestinal disorders, who maintained their habitual diet and had not used antibiotics for at least six months before donation.

To obtain a representative and homogeneous starting inoculum and minimize inter-individual variation, equal amounts of feces from each donor were pooled, thoroughly mixed, and diluted to 20% (*w*/*w*) with 0.1 M PBS under anaerobic conditions. The diluted slurry was filtered through double-layer sterile gauze, and the supernatant containing fecal microbiota was stored in an anaerobic chamber until use.

The basal medium was prepared according to the method described by Liu et al. [21]. Briefly, soluble starch (5 g), peptone (5 g), tryptone (5 g), yeast extract (4.5 g), NaCl (4.5 g), KCl (4.5 g), pectin (2 g), mucin (4 g), casein (3 g), NaHCO_3_ (1.5 g), L-cysteine hydrochloride (0.8 g), MgSO_4_·7H_2_O (1.23 g), guar gum (1 g), KH_2_PO_4_ (0.5 g), K_2_HPO_4_ (0.5 g), bile salts (0.4 g), CaCl_2_ (0.11 g), Tween 80 (1 mL), and resazurin (0.0111 g) were dissolved in ultrapure water. The solution was adjusted to pH 7.0, autoclaved at 121 °C for 20 min, and stored until use.

To construct the in vitro fermentation system, 0.5 g of lyophilized intestinal digesta residue powder was mixed with 2 mL of 0.1 M PBS, 5 mL of basal medium, and 5 mL of fecal slurry. Fermentation was conducted for 24 h, with sampling performed at 0.3, 2, 4, 8, 16, and 24 h by terminating the reaction at each time point.

Each extraction procedure was performed in triplicate, and all digestion and fermentation experiments were carried out under strictly anaerobic conditions. Upon completion, samples were immediately frozen in liquid nitrogen to halt biological activity and then stored at −80 °C for subsequent analysis. For gut microbiota and short-chain fatty acid analyses, fresh fecal slurry without any substrate served as the baseline control. Throughout the simulated gastrointestinal digestion and colonic fermentation, non-digested jackfruit powder was used as the control group (CG). A blank control without any sample was also included to eliminate background interferences from digestive fluids and the culture medium.

### 2.4. Characterization of Residues

The residues obtained from centrifugation of jackfruit powder and samples from the in vitro digestion and colonic fermentation processes were lyophilized for subsequent structural characterization.

#### 2.4.1. Scanning Electron Microscopy (SEM)

Following the method of Kenari and Razavi [22] with minor modifications, a small amount of jackfruit powder or residue was mounted on double-sided conductive carbon tape and examined in the SEM vacuum chamber under an acceleration voltage of 5 kV and a beam current of 10 mA. To improve electrical conductivity, the samples were sputter-coated with gold for 100 s before imaging.

#### 2.4.2. Fourier-Transform Infrared Spectroscopy (FTIR)

Following the method of Jiang et al. [23], FTIR analysis was conducted using the KBr pellet technique. An appropriate amount of jackfruit powder or residue was mixed and finely ground with KBr powder at a ratio of 1:100 to prepare pellets. The Frontier spectrometer was used to scan the samples over the wavenumber range of 4000–400 cm^−1^, with 64 scans per cycle and a spectral resolution of 4 cm^−1^. All spectral data were collected and processed using OMNIC software version 8.0, and each experiment was performed in triplicate to ensure reproducibility.

### 2.5. Extraction of Phenolic Compounds

Following the extraction method of Chen et al. [24] with several modifications, free, conjugated, and bound phenolics were isolated from jackfruit powder or from the freeze-dried residues obtained after in vitro digestion and colonic fermentation.

#### 2.5.1. Free Phenolics (FPs)

Jackfruit powder or the freeze-dried powder obtained from in vitro digestion and colonic fermentation was accurately weighed, and an 80% acetone solution was added at a solid-to-liquid ratio of 1:50 (*w*/*v*). After ultrasound-assisted extraction (400 W, 30 min, room temperature), the mixture was centrifuged at 12,000 rpm for 15 min at 4 °C. The extraction was repeated twice, and the resulting extracts were combined. After rotary evaporation at 35 °C to remove acetone, the concentrate was subjected to three liquid–liquid extractions with ethyl acetate (1:1, *v*/*v*). The organic phases were combined and concentrated under reduced pressure, and residual solvents were removed by nitrogen blowing to yield free phenolics (FPs).

#### 2.5.2. Conjugated Phenolics (CPs)

The aqueous phase remaining after FP extraction was mixed with an equal volume of 2 M NaOH solution and hydrolyzed at room temperature for 4 h. The mixture was then acidified to pH 2.0 with 6 M HCl, followed by three liquid–liquid extractions with ethyl acetate (1:1, *v*/*v*). The combined organic phases were concentrated under reduced pressure and dried by nitrogen blowing as described above to obtain conjugated phenolics (CP).

#### 2.5.3. Bound Phenolics (BPs)

The residue remaining after acetone extraction and centrifugation was suspended in 2 M NaOH at a solid-to-liquid ratio of 1:50 (*w*/*v*) and subjected to alkaline hydrolysis at room temperature for 4 h. The mixture was then acidified to pH 2.0 with 6 M HCl, centrifuged, and the resulting supernatant was extracted three times with ethyl acetate (1:1, *v*/*v*). The combined organic phases were concentrated under reduced pressure and dried by nitrogen blowing to yield bound phenolics (BP).

Each extraction procedure was performed in triplicate, and all extracts were stored at −80 °C before analysis.

### 2.6. Polyphenol Content Determination

#### 2.6.1. Total Phenolic Content (TPC)

The total phenolic content was determined using the Folin–Ciocalteu method described by Wekre et al. [25]. Briefly, 100 μL of jackfruit polyphenol solution was mixed with 500 μL of 50% Folin–Ciocalteu reagent and incubated in the dark for 8 min. Subsequently, 400 μL of 7.5% Na_2_CO_3_ solution was added, followed by a further 1 h incubation in the dark. The absorbance was measured at 765 nm using a microplate reader. Gallic acid served as the standard, with concentrations ranging from 0.02 to 0.24 mg/mL (R^2^ = 0.9992). Results were expressed as milligrams of gallic acid equivalents per gram of dry jackfruit powder (mg GAE/g DW). All measurements were performed independently in triplicate.

#### 2.6.2. Total Flavonoid Content (TFC)

The total flavonoid content was determined using the aluminum nitrate colorimetric method described by Li et al. [26]. Briefly, 75 μL of 724.6 mM NaNO_2_, 150 μL of 266.6 mM Al(NO_3_)_3_, and 300 μL of 1 M NaOH were sequentially added to 150 μL of jackfruit polyphenol solution, with 5 min dark incubations between each addition. The absorbance was measured at 510 nm using a microplate reader. Quantification was performed using a rutin standard curve (0.05–0.8 mg/mL, R^2^ = 0.9996), and results were expressed as milligrams of rutin equivalents per gram of dry weight (mg RE/g DW). All measurements were performed independently in triplicate.

### 2.7. HPLC-HR TOF MS^2^ Characterization and HPLC-PDA Quantification

Jackfruit polyphenols were analyzed using an HPLC system (LC-30A, Shimadzu, Japan) equipped with a photodiode array (PDA) detector. A C_18_ chromatographic column was maintained at 30 °C, and the injection volume was set to 10 μL. The mobile phase consisted of acetonitrile (A) and 0.1% formic acid in water (B). The gradient elution program was as follows: 0–6 min, 15% A; 6–25 min, 35% A; 25–40 min, 50% A; 40–45 min, 80% A; and 45–50 min, 15% A. The flow rate was 0.8 mL/min, and detection was performed at 280 nm.

Further structural characterization of the jackfruit polyphenols was conducted using high-performance liquid chromatography coupled with time-of-flight high-resolution mass spectrometry (HPLC-HR TOF MS^2^, AB SCIEX TripleTOF™ 6600+, manufactured by SCIEX, Foster City, CA, USA). The system comprised an HPLC unit and a mass spectrometer equipped with an electrospray ionization (ESI) source. Samples were analyzed in both positive and negative ion modes. The mass scan range for MS^1^ and MS^2^ was 50–1250 *m*/*z*. The ion spray voltage was set at ±5500 V or ±4500 V, with a declustering potential of ±80 V, collision energy of ±30 V, and a dispersion voltage of 15 V. For positive and negative detection modes, source gas 1 was maintained at 50 psi, source gas 2 at 60 psi, the curtain gas at 35 psi, and the interface temperature at 550 °C.

Quantification was performed using calibration curves constructed from the corresponding analytical standards. Compound identification was carried out using PeakView 2.1 software, and structural confirmation was based on exact mass measurements (mass error < 3 ppm), MS/MS fragmentation patterns, and comparison with standard retention times. Quantitative analysis followed the external standard method (R^2^ > 0.999). All chromatographic data were processed for peak integration and normalization using MasterView™ software version 1.1.

### 2.8. Antioxidant Activity of Phenolic Compounds

#### 2.8.1. DPPH Radical Scavenging Activity

The DPPH radical scavenging activity was determined according to the method described by Nakagawa et al. [27]. One hundred microliters of jackfruit polyphenol solution was mixed with 800 μL of 0.159 mM DPPH in ethanol. After incubation in the dark for 30 min, the absorbance was measured at 517 nm using a microplate reader. Quantification was performed using a Trolox standard curve (0.005–0.12 mg/mL, R^2^ = 0.9992), and results were expressed as Trolox equivalent concentrations (mg TE/mL). Each determination was performed independently in triplicate.

#### 2.8.2. ABTS Radical Scavenging Activity

The ABTS radical scavenging activity was determined according to the method described by Zhang et al. [28]. Ten milliliters of 7 mM ABTS stock solution was mixed with 176 μL of 140 mM K_2_S_2_O_8_ solution and incubated in the dark at 4 °C for 16 h to generate ABTS radicals. Before use, the solution was diluted with distilled water to an absorbance of 0.70 ± 0.02 at 734 nm to obtain the ABTS working solution. Eighty microliters of jackfruit polyphenol solution were then mixed with 800 μL of the ABTS working solution. After incubation in the dark for 6 min, the absorbance was measured at 734 nm using a microplate reader. Quantification was performed using a Trolox standard curve (0.005–0.12 mg/mL, R^2^ = 0.9993), and results were expressed as Trolox equivalent concentrations (mg TE/mL). Each determination was performed independently in triplicate.

#### 2.8.3. FRAP Reducing Power Assay

The FRAP working solution was freshly prepared according to the method described by Bouslamti et al. [29] by mixing 300 mM acetate buffer (pH 3.6), 10 mM TPTZ, and 20 mM FeCl_3_ at a volume ratio of 10:1:1, followed by pre-equilibration at 37 °C. One hundred microliters of jackfruit polyphenol solution was mixed with 900 μL of the FRAP working solution and incubated in the dark at 37 °C for 30 min. The absorbance was then measured at 593 nm using a microplate reader. The ferric ion reducing power was calculated using a Trolox standard curve (0.005–0.12 mg/mL, R^2^ = 0.9991), and results were expressed as mg TE/mL. Each determination was performed independently in triplicate.

### 2.9. Enzyme Inhibition Assays

#### 2.9.1. α-Glucosidase Inhibition Activity

The α-glucosidase inhibition assay was performed according to the method described by Gong et al. [30]. An α-glucosidase solution (0.04 U/mL in 200 mM PBS, pH 6.8) was mixed with jackfruit polyphenol solutions at various concentrations (0.011, 0.027, 0.086, 0.129, 0.171, 0.214 mg/mL) at a 2:1 volume ratio to obtain a final volume of 300 μL. After pre-incubation at 37 °C for 10 min, 200 μL of 5 mM PNPG substrate was added to initiate the reaction, which was terminated after 20 min by adding 0.2 M Na_2_CO_3_. The formation of p-nitrophenol was measured at 405 nm using a microplate reader.

#### 2.9.2. Pancreatic Lipase Inhibition Activity

The pancreatic lipase inhibition assay was conducted according to the method described by Zhou et al. [31], with slight modifications. The enzyme working solution (50 U/mL) was prepared in 50 mM Tris–HCl buffer containing 0.1% gum arabic and 0.2% sodium cholate (pH 7.1). The substrate system consisted of 1.5 mM PNPP containing 0.8% isopropyl alcohol and 0.2% Triton X-100. Eighty microliters of jackfruit polyphenol solutions at different concentrations (0.201, 0.818, 1.636, 2.16, 2.88, 3.6 mg/mL) were pre-incubated with 100 μL of the enzyme solution at 37 °C for 10 min, followed by the addition of 600 μL of substrate to initiate the reaction. The mixture was then incubated at 37 °C for 60 min, and the absorbance was subsequently measured at 405 nm.

#### 2.9.3. Xanthine Oxidase Inhibition Activity

The xanthine oxidase inhibition assay was conducted according to the method described by Dolati et al. [32]. Xanthine oxidase was dissolved in 50 mM Tris buffer (pH 7.5) to obtain a working solution at 0.1 U/mL. Jackfruit polyphenol solutions at various concentrations (0.088, 0.176, 0.263, 0.351, 0.439, 0.527 mg/mL) were vortexed with an equal volume of the enzyme solution and pre-incubated at 37 °C for 10 min. Then, 400 μL of 0.5 mM xanthine was added to initiate the reaction, giving a final volume of 600 μL, followed by incubation at 37 °C for 30 min. The reaction was terminated by adding 300 μL of 1 M HCl, and the absorbance was measured at 290 nm.

### 2.10. 16S rRNA Sequencing

Genomic DNA was extracted from fermentation samples using the cetyltrimethylammonium bromide (CTAB) method, and DNA concentration was adjusted to 1 ng/µL following verification by agarose gel electrophoresis. The bacterial 16S rRNA V4 region was amplified using barcoded primers 515F/806R and Phusion^®^ High-Fidelity PCR Master Mix with GC Buffer (New England Biolabs, Ipswich, MA, USA). PCR products were confirmed by 2% agarose gel electrophoresis, purified using magnetic beads, and pooled at equimolar concentrations. Size selection was performed via gel excision, and sequencing libraries were prepared using the TruSeq^®^ DNA PCR-Free Sample Preparation Kit (Illumina, San Diego, CA, USA). Libraries passing Qubit and qPCR quality checks were sequenced on an Illumina NovaSeq 6000 platform (Illumina, San Diego, CA, USA) using paired-end chemistry.

Raw reads were demultiplexed and stripped of barcode and primer sequences. Quality control was conducted using fastp (v0.23.2), removing reads containing ambiguous bases (N), reads with >40% low-quality bases, reads with sliding-window Phred quality < 20, reads containing polyG tails, and reads shorter than 150 bp. High-quality paired-end reads were merged using FLASH (v1.2.11), and unmerged reads were discarded. Chimeric sequences were detected and removed using vsearch, and the resulting high-quality merged reads were retained as effective tags for downstream analyses. Per-sample counts of raw tags, clean tags, effective tags, and associated quality metrics are summarized in Table S1.

Bioinformatics processing was performed in QIIME 2 (v2024.5). Denoising, ASV inference, and chimera removal were conducted using the DADA2 plugin implemented in QIIME 2 with default parameters. Representative ASV sequences were taxonomically assigned using the Mothur classifier against the SILVA SSU rRNA database (v138.1), with a confidence threshold of 0.8–1.0. Multiple sequence alignment of ASV representative sequences was performed using MAFFT (v7.525), followed by phylogenetic tree construction.

To ensure comparability across samples, read counts were normalized by rarefaction to the minimum sequencing depth observed among samples. All downstream α-diversity (Observed Species, Chao1, ACE, Shannon, Simpson) and β-diversity analyses were conducted on the normalized dataset.

All raw sequencing reads have been deposited in CNCB (https://ngdc.cncb.ac.cn/gsa/s/jl5XZoXC, accessed on 15 September 2025), enabling full reproducibility of the analytical workflow.

### 2.11. Determination of Short-Chain Fatty Acid (SCFA) Content

The colonic fermentation broth was thawed and vortex-mixed. Then, 50 μL of the sample was transferred into a 1.5 mL centrifuge tube and vortex-mixed with 100 μL of 0.5% (*v*/*v*) phosphoric acid solution. Subsequently, 750 μL of methyl tert-butyl ether (MTBE) extractant containing internal standards was added. After thorough vortex-mixing, the mixture was subjected to ultrasonication at 4 °C for 5 min and then centrifuged at 12,000 r/min for 10 min at 4 °C. After centrifugation, 200 μL of the supernatant was transferred into a vial insert for analysis.

SCFA analysis was performed using a gas chromatography–tandem mass spectrometry (GC–MS/MS) system. The injection volume was set to 1 μL in split mode (split ratio 5:1), with helium as the carrier gas at a flow rate of 1.2 mL/min. Separation was achieved on a DB-FFAP capillary column (30 m × 0.25 mm × 0.25 μm) using the following temperature program: the column was held at 50 °C for 1 min, ramped to 220 °C at 18 °C/min, and maintained for 5 min. The injection port and transfer line temperatures were set to 250 °C and 230 °C, respectively. The ion source and quadrupole temperatures were maintained at 230 °C and 150 °C, respectively, and the electron-impact ionization energy was set to 70 eV. Data were acquired in dynamic multiple reaction monitoring (dMRM) mode with a solvent delay of 5 min.

### 2.12. Statistical Analysis

All measurements were performed in triplicate, and the results are expressed as mean ± standard deviation. The Shapiro–Wilk test was used to assess the normality of the data, and Levene’s test was applied to evaluate the homogeneity of variances before statistical analysis. For datasets meeting both normality and homogeneity assumptions, one-way analysis of variance (ANOVA) was conducted to evaluate the overall effects of the ripening stages. When ANOVA indicated significant differences (*p* < 0.05), Duncan’s multiple range test was applied for pairwise comparisons among groups. Data that did not follow a normal distribution were analyzed using the non-parametric Kruskal–Wallis H test. The significance level for all statistical tests was set at *p* < 0.05. All statistical analyses were performed using IBM SPSS Statistics software (version 27.0; IBM Corp., Armonk, NY, USA).

## 3. Result and Discussion

### 3.1. Structural Characterization of Residues

#### 3.1.1. SEM

**Figure 1:**
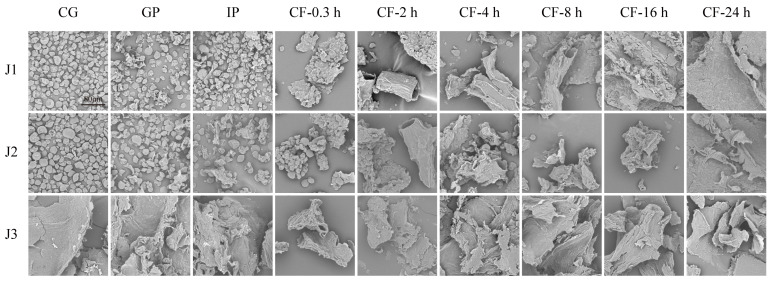
SEM micrographs of residues collected during in vitro gastrointestinal digestion and subsequent colonic fermentation of jackfruit at distinct ripening stages (scale bar = 80 µm). J1, J2, and J3 represent jackfruit samples of increasing ripeness; CG denotes the control group; GP and IP refer to the gastric and intestinal phases, respectively; and CF indicates the colonic fermentation phase, with samples collected at 0.3, 2, 4, 8, 16, and 24 h.

In this study, a scanning electron microscope was used to examine the morphological evolution of jackfruit residues with different maturity levels during in vitro digestion and colonic fermentation. In the undigested stage, J1 and J2 exhibited irregular oval shapes, while J3 displayed a curled, sheet-like structure (Figure 1). This difference can be attributed to dynamic changes in the nutritional composition of jackfruit flesh during maturation. Early in maturation, starch content is relatively high, whereas soluble sugar increases markedly in later stages [3]. Different nutrients exhibit distinct morphologies under scanning electron microscopy. Although the oral phase (OP) was included in the experiment, its effect on polyphenols is negligible [33] and is therefore not discussed further.

In the gastrointestinal phase, digestive enzymes (pepsin and trypsin) hydrolyze the cell wall matrix, causing minor surface disruption and partial degradation of the protein framework and polysaccharide network [34]. During colonic fermentation (CF), J1 and J2 residues underwent a series of morphological transitions, including clustered aggregates, tubular structures with distinct fiber textures, roughened tubular surfaces, pasty masses, and eventually smooth lamellar structures. These changes result from targeted hydrolysis of lignin–polyphenol complexes by cellulases secreted by colonic microbiota, which promotes the release of fiber-bound polyphenols [35]. The final smooth lamellar structure indicates the complete breakdown of the microstructure of jackfruit residues. In contrast, J3 residues showed only minor changes in surface micro-roughness throughout the process. Although no macro-scale disintegration was observed, the results still confirm the degradative effects of digestive enzymes and microorganisms on the matrix.

In summary, scanning electron microscopy revealed that digestive enzymes and colonic microbiota work together to degrade jackfruit residues.

#### 3.1.2. FTIR

**Figure 2:**
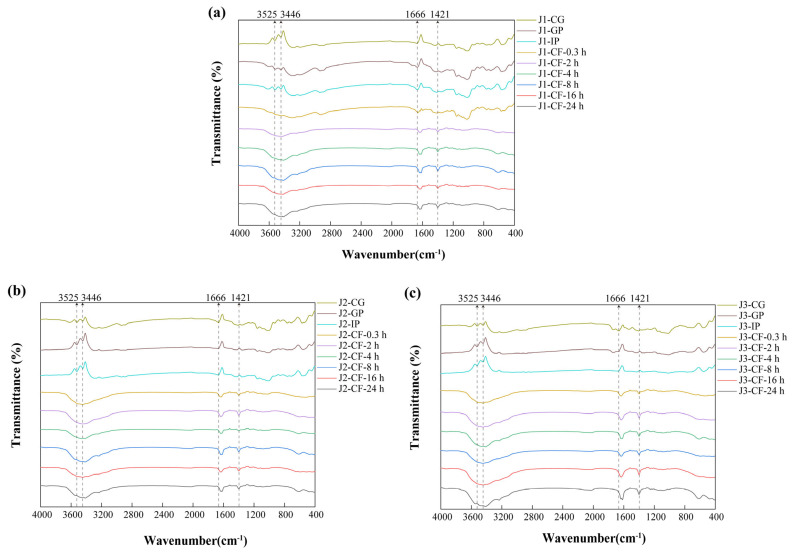
FTIR characterization of residues obtained during in vitro digestion and colonic fermentation of jackfruit at different maturity levels. (**a**) J1, (**b**) J2, and (**c**) J3. J1, J2, and J3 represent jackfruit samples of increasing ripeness; CG denotes the control group; GP and IP refer to the gastric and intestinal phases, respectively; and CF indicates the colonic fermentation phase, with samples collected at 0.3, 2, 4, 8, 16, and 24 h.

FTIR analysis can identify differences in the functional groups and structural features of phenolic compounds among samples [36]. The similarity in FTIR spectra of jackfruit at different maturity stages (Figure 2) suggests that maturation mainly affects the content of polyphenols and intermolecular interactions, while the structural integrity of key functional groups in bound polyphenols, such as hydroxyl groups and aromatic rings, remains unchanged. The absorption peak at 3525 cm^−1^ corresponds to intramolecular hydrogen bonds of phenolic hydroxyl groups, whereas the peak at 3446 cm^−1^ is associated with intermolecular hydrogen bonds [37]. The stability of these peaks during the gastrointestinal phase indicates that gastric acid and proteases cause only limited disruption of the covalent bonds associated with phenolic hydroxyl groups. In contrast, the gradual decrease in peak intensity during colonic fermentation is attributed to microbial secretion of phenolic acid decarboxylases and ring-cleaving enzymes, which convert phenolic hydroxyl groups into quinoid structures or aliphatic carboxyl groups, thereby disrupting the hydrogen-bond network.

The absorption peak at 1666 cm^−1^ is assigned to the carbonyl stretching vibrations of bound phenolics, such as polysaccharide–phenolic acid ester bonds present in cell wall matrices. During colonic fermentation, the increased intensity and blue shift in this peak reflect two microbial processes. First, esterase-mediated hydrolysis of ester bonds releases free phenolic acids and exposes additional carbonyl groups. At the same time, high-molecular-weight polyphenols are degraded into low-molecular-weight derivatives whose carboxylic acid carbonyl peaks (1720–1700 cm^−1^) overlap with residual ester-bond signals, thereby contributing to the observed blue shift [38]. The peak at 1421 cm^−1^ primarily arises from C=C skeletal vibrations of aromatic rings and secondarily from in-plane deformation vibrations of O–H in phenolic hydroxyls. Its increased intensity during colonic fermentation is attributed to dissociation of lignin–polyphenol complexes, which removes shielding of aromatic ring signals, and to deprotonation of phenolic hydroxyl groups, which enhances O–H deformation vibrations [39].

Overall, FTIR analysis complements SEM observations by revealing structural changes at the molecular-bonding level and provides a theoretical basis for improving the bioavailability of polyphenols through targeted modulation of ester-bond and hydrogen-bond networks.

### 3.2. Polyphenol Content

**Figure 3:**
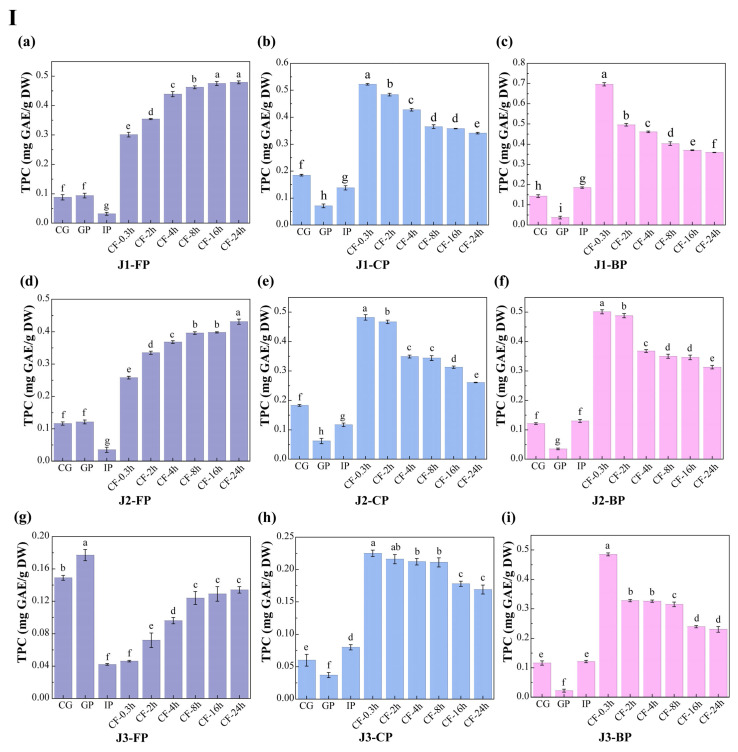

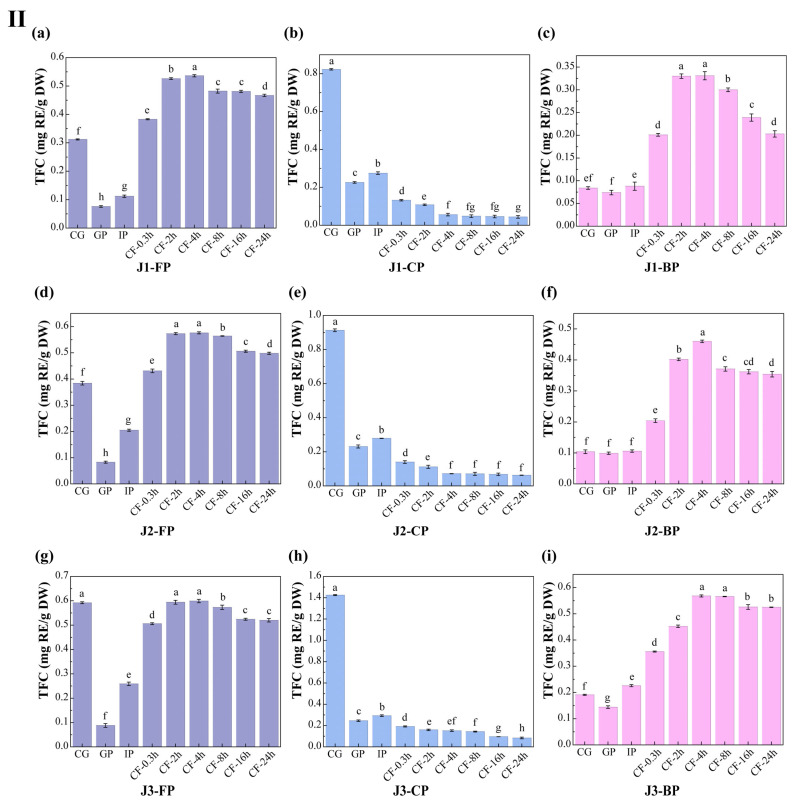
Changes in polyphenol content in jackfruit of different maturity stages during in vitro simulated digestion and colonic fermentation. (**I**) denotes total phenolic content (TPC), and (**II**) denotes total flavonoid content (TFC). (**a**–**c**) correspond to the FP, CP, and BP fractions of jackfruit at the J1 maturity stage; (**d**–**f**) correspond to the FP, CP, and BP fractions at the J2 stage; and (**g**–**i**) correspond to the FP, CP, and BP fractions at the J3 stage. FP, CP, and BP represent free, conjugated, and bound polyphenols, respectively. J1, J2, and J3 refer to jackfruit samples of increasing ripeness. CG denotes the control group; GP and IP represent the gastric and intestinal phases, respectively; and CF represents the colonic fermentation phase, with samples collected at 0.3, 2, 4, 8, 16, and 24 h. Different lowercase letters in the figure indicate statistically significant differences among groups.

The dynamic changes in total polyphenol content (TPC) and total flavonoid content (TFC) in free polyphenols (FP), conjugated polyphenols (CP), and bound polyphenols (BP) of jackfruit at different maturity stages during in vitro digestion and colonic fermentation were measured using the Folin–Ciocalteu and Al(NO_3_)_3_ methods (Figure 3).

During the undigested stage, FP-TPC increased during ripening, while CP-TPC and BP-TPC decreased. These changes were primarily due to the synergistic action of endogenous glycosidases, esterases, and cell-wall degradation enzymes. This finding aligns with Mokhtar et al. [40], who reported that the free phenolic content in pumpkin increases with maturity. TFC in all polyphenol forms also increased with maturity. FP-TFC increased for reasons similar to those driving the rise in FP-TPC. The increase in CP-TFC may result from the upregulation of the UGT78D2 gene during maturation, which enhances flavonoid glycosylation [41]. The increase in BP-TFC can be attributed to stronger flavonoid-cell-wall binding (as seen in the FTIR analysis, where the characteristic peak at 1666 cm^−1^ intensified in Figure 2) and specific deposition of hydroxycinnamic acid-esterified flavones during lignification.

Polyphenol changes during in vitro digestion and fermentation depend on their binding forms, with similar trends across different maturity stages. In the gastric phase (GP), FP-TPC increased, while CP-TPC and BP-TPC decreased. Gastric acid broke ester and glycosidic bonds, and pepsin degraded polyphenol-protein complexes, promoting conversion of CP and BP to FP. During this phase, TFC in all forms dropped significantly. BP-TFC decreased as pepsin indirectly converted part of it to CP and FP. CP-TFC was degraded in the gastric environment, degradation exceeded generation, resulting in a net reduction in CP-TFC. FP-TFC also declined due to oxidation in the acidic stomach, as flavonoids, with their multi-hydroxyl and conjugated double bonds, are prone to oxidation.

In the intestinal phase (IP), hydrophobic polyphenols form soluble micellar conjugates with bile salts, while quinone oxidation products covalently crosslink with dietary fiber to form insoluble complexes. Additionally, pancreatic enzymes hydrolyze hydrophobic ester bonds, generating new conjugates [42]. These processes caused FP-TPC to decrease, while CP-TPC and BP-TPC increased. However, TFC in all forms trended upward in this phase: FP-TFC rose significantly; CP-TFC temporarily accumulated as its formation outpaced hydrolysis; BP-TFC increased temporarily as hydrolysis converted it into more detectable small molecules.

In the colonic fermentation (CF) phase, microbial metabolism dominates polyphenol transformation. Microbial enzymes catalyze hydrolysis and demethylation of CP and BP, releasing FP and converting complex precursors into small-molecule phenolic acids. As a result, FP-TPC increased, while CP-TPC and BP-TPC decreased. For TFC, during early CF, extensive enzymatic hydrolysis released free flavonoid aglycones. Release exceeded microbial metabolism, leading to a rise in FP-TFC. In late CF, microbes extensively degraded all flavonoid forms via C-ring cleavage and dehydroxylation, converting them into phenylpropanoid derivatives, gases, and short-chain fatty acids (SCFAs) (Table S6 shows an increase in SCFA production), causing TFC in all forms to decline.

In summary, this study provides key theoretical insights into the mechanisms of polyphenol transformation in complex plant matrices during in vitro digestion and fermentation.

### 3.3. HPLC-HR TOF MS2 Characterization and HPLC-PDA Quantification of Polyphenols

Untargeted HPLC–HR TOF MS^2^ of jackfruit pulp revealed 263 differential features (VIP > 1, *p* < 0.05), tentative annotation as phenolic acids and flavonoids. Guided by these untargeted results, we subsequently quantified 18 representative phenolic compounds (nine phenolic acids and nine flavonoids) using a validated external-standard method (R^2^ ≥ 0.99 for all calibration curves) (Table 1). Detailed quantification is given in Tables S2–S4.

FP-gallic acid (GA) showed no significant differences among the three maturity stages in the undigested phase and peaked in the gastric phase (GP). This was attributed to gastric acid promoting CP hydrolysis and increasing FP content. FP-caffeic acid (CA) remained stable in the gastrointestinal phase and significantly increased in the colonic fermentation (CF) phase. FP-chlorogenic acid (CGA) increased in CF as microbial glycosidase and esterase catalyzed breakdown of BP and residual CP. FP-naringenin (Nar) was only detected in the maturity stage J1 and was absent in J2/J3. Zheng et al. [43] found that Nar synthase genes are highly expressed early and down-regulated later, while catabolic enzyme genes show the opposite trend, indicating maturation-dependent Nar metabolism. FP-epicatechin gallate (ECG) was undetectable during gastrointestinal digestion but appeared in CF. It might be generated via microbial enzyme hydrolysis of CP or transformation of BP.

CP-GA peaked in the intestinal phase (IP) as alkaline fluid promoted trypsin hydrolysis of hydrophobic ester bonds [42]. CP-quercetin (QCT) was only detected in the gastrointestinal phase of all maturity stages. In CF, microbial hydrolysis of glycosidic bonds converted it into the free form, rendering CP undetectable. CP-taxifolin (Tax) was absent in J1 but present in J2 and J3. J2 and J3 may upregulate glycosyltransferase activity to promote glycosylation and form CP-Tax [41], while J1 lacks this capacity.

BP-caffeic acid (CA), homovanillic acid (HVA), ECG, Tax, and QCT were undetectable during gastrointestinal digestion of J1 and J2 but appeared in CF, possibly due to microbial formation of new BPs. BP-hyperoside (Hyp), glycitein (Gly), and mulberrin (Mul) were absent in J1 but present in J2 and J3. Physiological and biochemical reactions in later maturation promote their synthesis and transformation, processes not yet initiated in J1.

In summary, this study systematically elucidates the metabolic transformation patterns of jackfruit polyphenols at different maturity stages during digestion, providing a theoretical basis for precisely regulating the harvest window to enhance the bioavailability of dietary polyphenols.

### 3.4. Antioxidant Activity

The biological relevance of polyphenols within the gastrointestinal tract primarily depends on the bioaccessible fraction [44]. Measuring antioxidant activity during in vitro digestion is essential to evaluate the antioxidant potential of polyphenols within the gastrointestinal lumen. This approach allows dynamic tracking of polyphenol release, degradation, and structural reorganization across the gastric–intestinal–colonic continuum under simulated digestive conditions. It therefore assesses the “bioaccessible antioxidant potential” retained in the lumen after digestion, avoiding bias from relying solely on the antioxidant capacity of undigested samples. Moreover, changes in antioxidant activity during digestion provide insights into how structural transformations influence antioxidant behavior in the intestinal milieu. These changes help identify the structures or metabolites that contribute most to antioxidant effects in the lumen. In addition, these results provide a basis for interpreting the potential roles of polyphenols in modulating the redox environment of the intestinal contents and shaping luminal conditions relevant to microbial activity. However, in vitro models are simplified systems and cannot fully reflect the complexity of in vivo metabolism. Therefore, these findings should be considered indicative of antioxidant potential in the lumen, and further in vivo studies are required to evaluate bioavailability and bioactivity beyond the gastrointestinal tract.

**Figure 4:**
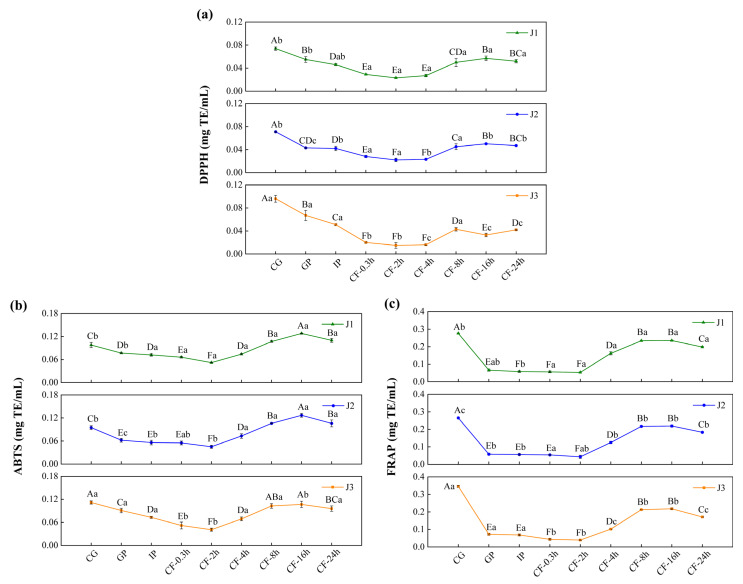
Determination of the antioxidant activity of bioaccessible polyphenols in jackfruit of different maturity levels during in vitro digestion and colonic fermentation: (**a**) DPPH, (**b**) ABTS, and (**c**) FRAP. J1, J2, and J3 represent jackfruit samples of increasing ripeness; CG denotes the control group; GP and IP refer to the gastric and intestinal phases, respectively; and CF indicates the colonic fermentation phase, with samples collected at 0.3, 2, 4, 8, 16, and 24 h. Different lowercase letters indicate significant differences in polyphenol antioxidant activity among samples of different maturity at the same digestion stage, while different capital letters denote significant differences across digestion stages within the same maturity level (*p* < 0.05).

In the undigested and gastrointestinal phases, the antioxidant activity of jackfruit polyphenols increased steadily with increasing ripeness (Figure 4). This increase was associated with the accumulation of low-molecular-weight phenolic acids and free flavonols in the late ripening stage. By donating hydrogen atoms or electrons, these compounds enhanced radical scavenging and ferric-ion reducing power. However, this trend was reversed during colonic fermentation (CF). In high-maturity jackfruit, which contained abundant free phenolics, these compounds were rapidly consumed or converted into inert metabolites during early fermentation, leading to a depletion of antioxidant capacity. In contrast, low-maturity jackfruit, which was rich in bound phenolics, converted them into active small-molecule phenolic acids via microbial degradation.

Across all maturity levels, jackfruit exhibited similar changes in antioxidant activity during digestion. In the gastric phase (GP), antioxidant activity declined, consistent with the variation trend of FP-TFC, indicating degradation of bioactive flavonoids in the acidic gastric environment. In the intestinal phase (IP), antioxidant activity declined further owing to the oxidation and degradation of phenolic compounds in the neutral to mildly alkaline intestinal environment [45]. During the CF phase, antioxidant activity first increased and then decreased. The initial increase was attributed to structural modifications of phenolic compounds, specifically transformation of bound phenolics into more bioactive free forms. The subsequent decline was mainly due to two factors: depletion of bound-phenolic reserves through prior conversion and further microbial metabolism of released free phenolics into inactive end products [46].

In conclusion, the antioxidant capacity of jackfruit during digestion is primarily determined by the bioaccessible fraction of its polyphenols within the gastrointestinal lumen, particularly in the colonic phase. Fruit ripeness and gastrointestinal transit jointly regulate the release, transformation, and depletion of luminal phenolic compounds, thereby shaping the antioxidant environment of the intestinal contents. As bioavailability and systemic absorption of phenolic metabolites were not assessed in this study, further in vivo investigations are required to elucidate their potential bioactivity beyond the gastrointestinal tract.

### 3.5. Enzyme Inhibitory Activity

**Figure 5:**
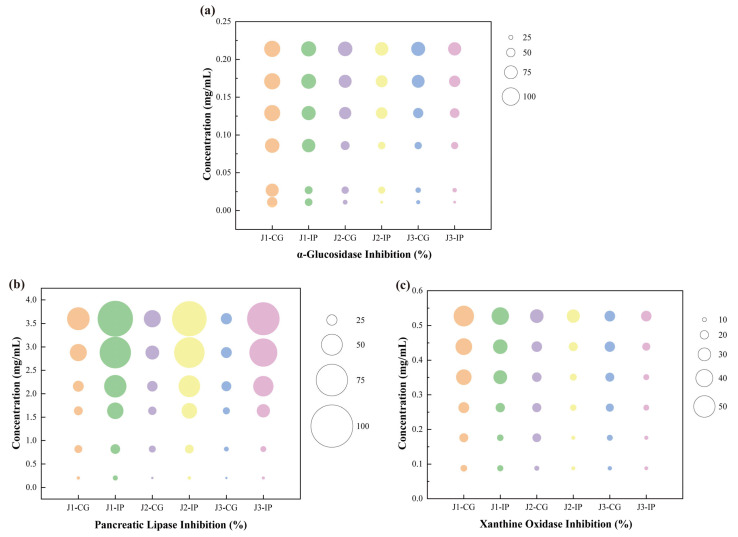
Determination of key enzyme inhibitory activities of bioaccessible polyphenols released from jackfruit at different ripening stages following simulated intestinal digestion: (**a**) α-glucosidase inhibition, (**b**) pancreatic lipase inhibition, and (**c**) xanthine oxidase inhibition. J1, J2, and J3 represent jackfruit samples of increasing ripeness; CG denotes the control group;IP refer to the intestinal phases.

The key enzyme inhibitory activities of bioaccessible polyphenols released after simulated intestinal digestion of jackfruit at different ripening stages were evaluated. The inhibitory activities against α-glucosidase, pancreatic lipase, and xanthine oxidase were examined. α-Glucosidase and pancreatic lipase are essential intestinal digestive enzymes, whereas xanthine oxidase—although primarily hepatic—is influenced by intestinal metabolites and linked to hyperuricemia and gout [47]. As shown in Figure 5, the inhibitory effects of jackfruit polyphenols on these enzymes exhibited clear concentration dependence.

Compared with unripe fruit, higher ripeness reduced the inhibitory activities of jackfruit polyphenols against α-glucosidase, pancreatic lipase, and xanthine oxidase. The inhibitory activities of intestinal-phase (IP) polyphenols against α-glucosidase and xanthine oxidase decreased relative to the undigested samples, consistent with observations by Dou et al. [48] and Zhu et al. [49]. In contrast, the inhibitory activity against pancreatic lipase increased after digestion, similar to the trend reported by Gutiérrez-Grijalva et al. [50] for oregano polyphenols. These maturity- and digestion-associated shifts in enzyme inhibition appear to be driven by two primary mechanisms. First, fruit maturation and protease digestion substantially altered polyphenol composition (Table 1), thereby influencing inhibitory potency—an effect also reflected in the wide variability in α-glucosidase inhibition reported among 11 flavonoids by Fu et al. [51]. Second, the binding state of polyphenols changed dynamically during maturation and digestion. Protein–polyphenol complexes generally reduce inhibitory activity [52], whereas starch–polyphenol interactions show structure-dependent effects: amylose complexes often decrease activity, while amylopectin complexes may enhance it through steric hindrance [53].

To further contextualize the inhibitory potency of the jackfruit polyphenol mixture, its effective concentrations were compared with reported IC_50_ values of reference inhibitors—ferulic acid (0.866 mg mL^−1^), catechin (0.280 mg mL^−1^), and quercetin (8.327 μmol L^−1^) [54,55,56]. These comparisons indicate that, despite being a mixture, jackfruit polyphenols can achieve biologically relevant inhibitory concentrations of all tested enzymes and exhibit superior α-glucosidase inhibition, reinforcing their potential as a multifunctional food ingredient.

Given these inhibitory potencies, a key question is whether such concentrations are achievable through normal dietary intake. Although the in vitro effective concentrations are higher than eventual systemic plasma levels, they remain physiologically relevant because polyphenols reach high local concentrations in the gastrointestinal lumen. Thus, the inhibitory potential observed here reflects a biologically plausible activity following jackfruit consumption.

### 3.6. Colon Fermentation Pathway

**Figure 6:**
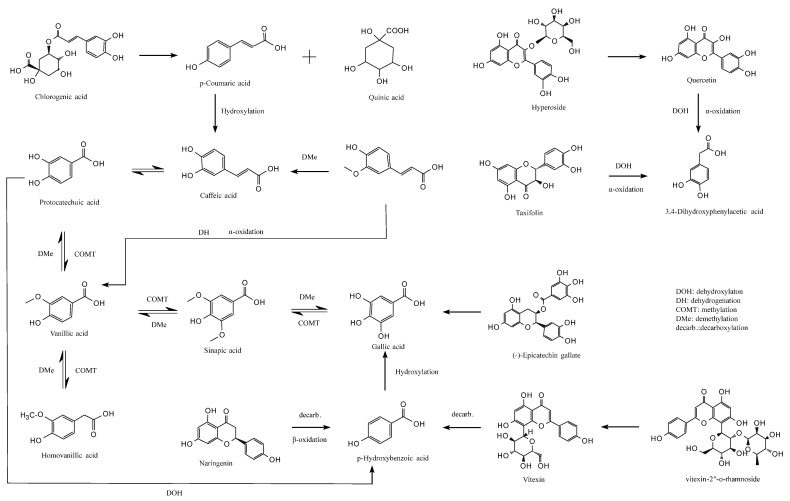
Proposed metabolic pathways of major jackfruit polyphenols during simulated colonic fermentation.

This study elucidates the potential metabolic pathways of major jackfruit polyphenols during colonic fermentation (Figure 6). During the colonic fermentation (CF) phase, microbial esterases hydrolyze the ester bonds of chlorogenic acid (CGA), generating p-coumaric acid (pCA) and quinic acid [57]. pCA can subsequently undergo C3-position hydroxylation catalyzed by microbial hydroxylases, forming caffeic acid (CA), which exhibits enhanced antioxidant activity [58]. In parallel, microbial demethylases selectively remove the methoxy group (–OCH_3_) from ferulic acid (FA), likewise converting it into CA [59]. In addition, the acrylic side chain of FA undergoes microbially mediated α-oxidation, including dehydrogenation and chain shortening, yielding vanillic acid (VA), a low-molecular-weight metabolite with greater colonic absorption [60].

VA participates in a dynamic equilibrium metabolism in which microbial methyltransferases catalyze benzene-ring methylation to form sinapic acid (SA), whereas SA can be reconverted to VA by dehydroxylases [61]. Microbial hydroxylases can also hydroxylate VA at the C5 position to generate homovanillic acid (HVA), which can be reversibly reduced to VA by dehydroxylases [59]. These bidirectional conversions modulate the polarity of phenolic acids and thereby influence their passive diffusion across the colonic epithelium. Furthermore, SA can undergo microbial demethylation to produce gallic acid (GA) with a pyrogallol-type trihydroxyl structure, whereas specific microbial strains can remethylate GA to regenerate SA [62]. VA may also be demethylated to form protocatechuic acid (PCA), which possesses a catechol structure; conversely, PCA can be remethylated to regenerate VA, enabling flexible regulation of phenolic acid redox properties [60].

PCA undergoes irreversible C2-position dehydroxylation catalyzed by microbial decarboxylases, forming p-hydroxybenzoic acid (pHBA) [63]. This degradation step simplifies the molecular structure of phenolic acids and enhances their absorption and utilization. pHBA may also undergo hydroxylation at the C3 and C5 positions, catalyzed by microbial hydroxylases to form GA [62], with additional hydroxyl groups strengthening its free-radical-scavenging capacity.

For flavonoids, microbial β-glucosidases hydrolyze the glycosidic bonds of hyperoside (Hyp), releasing the free flavonol quercetin (QCT) and improving bioactivity and intestinal uptake. QCT is subsequently cleaved at the C-ring (pyran ring) by microbial dioxygenases, generating a phenolic acid intermediate that, following dehydroxylation and α-oxidative carboxylation, is converted into 3,4-dihydroxyphenylacetic acid (DOPAC) [64]. A similar metabolic route occurs for taxifolin (Tax), which undergoes C-ring cleavage, dehydroxylation, and side-chain α-oxidation, ultimately yielding DOPAC, indicating that structurally distinct flavonoids can converge to a common final metabolite [65]. Additionally, microbial α-rhamnosidases hydrolyze the glycosidic bonds of vitexin-2″-O-rhamnoside (VR), releasing orientin, which then undergoes C-ring cleavage and decarboxylation to produce pHBA. Naringenin (Nar), following microbial C-ring cleavage, β-oxidation, and decarboxylation, is also converted into pHBA [66], converging with the metabolic fate of QCT-derived flavonols. Notably, microbial esterases specifically hydrolyze the ester bonds of epicatechin gallate (ECG), releasing free GA and epicatechin—representing a key step in colonic bioactivation of condensed tannins [67].

Overall, this study delineates the complex metabolic network governing the biotransformation of phenolic compounds during colonic fermentation. These findings provide a theoretical foundation for designing precise nutritional interventions, preventing and managing chronic diseases, and developing microbiota-targeted therapeutic strategies.

### 3.7. Changes in Intestinal Microorganisms During Simulated Colonic Fermentation

This study employed 16S rRNA gene amplicon sequencing to investigate how jackfruit of different maturity stages modulates the human gut microbiota in an in vitro colonic fermentation model. *Actinobacteria*, *Bacteroidetes*, *Firmicutes*, and *Proteobacteria* constituted the core phyla underpinning the structural and functional characteristics of the intestinal microbial community [35]. α-Diversity indices—including Observed Species, Chao1, ACE, Shannon, and Simpson—were calculated to assess within-sample richness and community evenness [68]. Observed Species reflects the number of detected OTUs; Chao1 and ACE estimate total richness including rare taxa; higher Shannon values indicate greater community evenness, whereas higher Simpson values denote dominance by a few taxa and thus lower diversity (Table S5).

**Figure 7:**
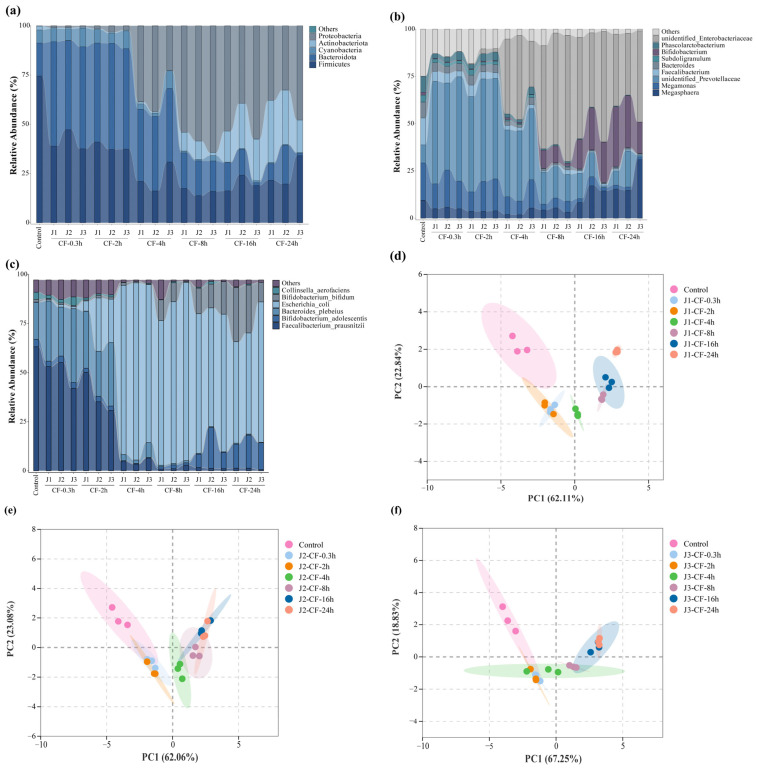
Effects of jackfruit on colonic microbiota during in vitro colonic fermentation. (**a**–**c**) Analysis of microbial community richness at the phylum, genus, and species levels, the category “Others” represents taxa with very low relative abundance and is shown as a thin layer at the top of each stacked bar; (**d**–**f**) principal component analysis (PCA), the shaded areas represent the 95% confidence ellipses of the biological replicates in the PCA score plot.

*Actinobacterial* abundance increased markedly along the maturity gradient (J1 > J2 > J3) (Figure 7a–c). This pattern paralleled the decline in bound-phenolic total phenolic content (BP-TPC) during ripening, suggesting a positive association between *Actinobacterial* enrichment and the elevated bound-phenolic load of pre-climacteric fruit. Microbial de-esterification during colonic fermentation released free phenolics (e.g., caffeic acid, taxifolin, and vitexin-rhamnoside), which selectively stimulated the growth of *Bifidobacterium* spp.—a trend that waned as BP levels declined during maturation.

In addition to *Bifidobacterium*, *Megasphaera* exhibited a gradual increase, whereas *Megamonas* declined throughout fermentation. A concomitant reduction in *unidentified Prevotellaceae* indicated targeted suppression of phenolic-sensitive taxa. Conversely, a slight rise in *unidentified_Enterobacteriaceae* was observed, although its contribution to the overall community structure remained limited.

At the phylum level, the reduction in *Bacteroidetes* reflected the decreasing abundance of *Prevotellaceae*-related genera, while *Firmicutes* transiently declined in early fermentation—likely owing to phenolic inhibition—before rebounding as phenolic transformation reshaped the community structure and supported the proliferation of butyrate-associated taxa such as *Faecalibacterium*. *Proteobacteria* also showed a significant decrease.

Principal component analysis (PCA) revealed a clear separation between the fresh fecal inoculum and all treatment groups (Figure 7d–f), attributable to rapid loss of host-restricted taxa deprived of host-derived factors outside the host environment. Samples from different maturity stages further diverged along the temporal axis, underscoring the regulatory influence of jackfruit polyphenols on colonic microbiota.

Collectively, these findings demonstrate that jackfruit polyphenols selectively promote beneficial taxa while suppressing potential pathogens, thereby enhancing intestinal barrier function. However, although the pooled-sample design ensured a homogeneous inoculum and minimized technical variation, it also limited capture of natural inter-individual microbiome variation. Consequently, the conclusions of this study apply only to this specific synthetic community, and extrapolation to broader populations remains constrained. Future studies incorporating independent biological replicates will be necessary to establish the generalizability of these observed effects.

### 3.8. SCFAs

**Figure 8:**
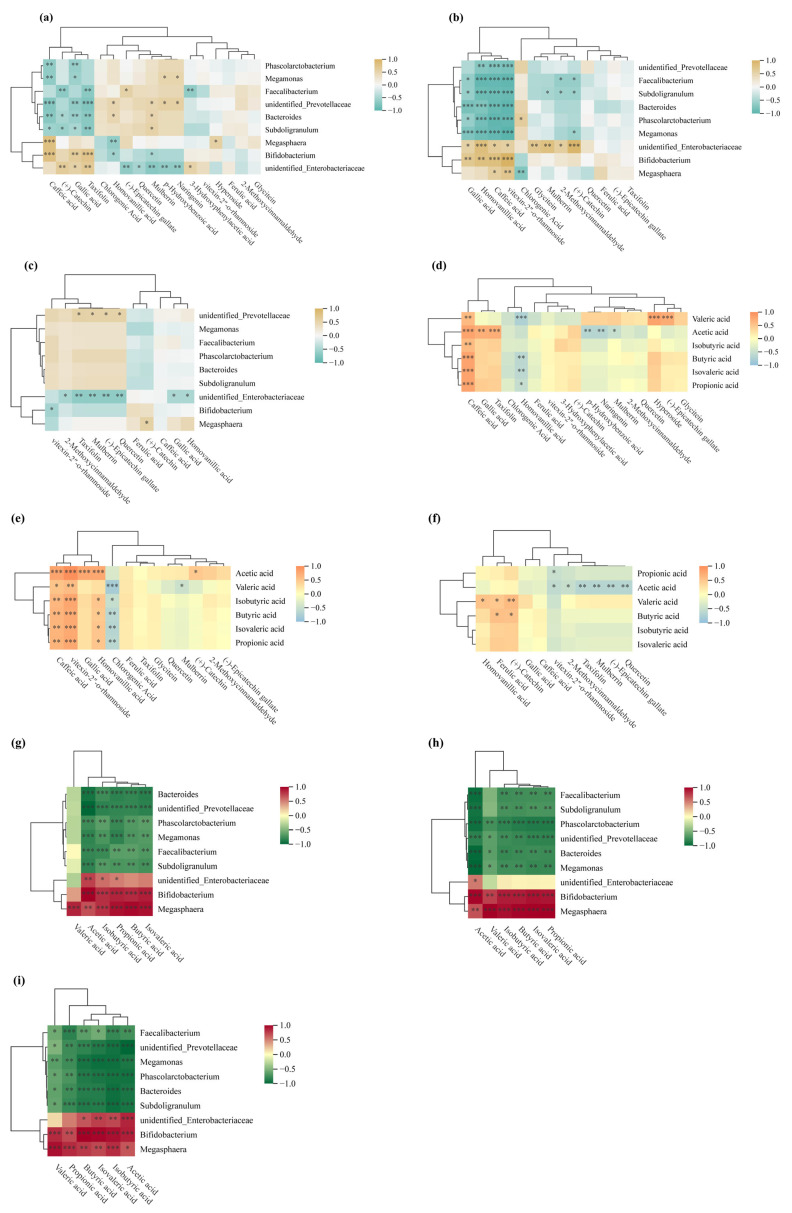
Spearman correlation analysis. (**a**–**c**) Spearman correlation heatmaps between polyphenols and the gut microbiome at the genus level; (**d**–**f**) Spearman correlation heatmaps linking polyphenols to short-chain fatty acids (SCFAs); (**g**–**i**) Spearman correlation heatmaps between the gut microbiome and SCFAs. * *p* < 0.05; ** *p* < 0.01; *** *p* < 0.001.

Gut microbiota ferment gastrointestinal-derived undigested residues, producing short-chain fatty acids (SCFAs), primarily acetate, propionate, and butyrate [69]. Released phenolic compounds and their metabolites (e.g., caffeic acid, gallic acid, vitexin-2″-O-rhamnoside, homovanillic acid, and taxifolin) selectively promote growth of beneficial bacteria such as *Bifidobacterium*, *Lactobacillus*, *Faecalibacterium prausnitzii*, and *Roseburia recti* (Figure 8a–c). As major SCFA producers, these bacteria are enhanced by phenolic substances that inhibit harmful bacterial proliferation, thereby markedly enhancing SCFA synthesis efficiency [35]. Therefore, colonic fermentation degrades jackfruit-bound phenolics, releases bioactive molecules and reshapes the microbiota to favor beneficial proliferation and efficient SCFA production (Figure 8d–f).

In vitro fermentation showed SCFA yields increased continuously over time for jackfruit at all ripeness stages (Table S6), following the order J1 > J2 > J3. Correlation analysis revealed several dominant genera strongly associated with this SCFA accumulation trend (Figure 8g–i): *Bifidobacterium* and *Bacteroides* were positively correlated with acetate production, *Phascolarctobacterium* showed a pronounced positive relationship with propionate, while *Megasphaera* demonstrated the strongest association with butyrate formation. Conversely, *Megamonas* and *unidentified Prevotellaceae* showed weak or negative correlations with total SCFA levels. These microbial–metabolite linkages indicate that the fermentation-driven restructuring of the gut microbiota played a key role in shaping the SCFA output profile across maturity stages.

This pattern links to structural and compositional changes in the cell wall during fruit development. J1 residues possess denser and more rigid cell walls enriched in structural polysaccharides such as cellulose and hemicellulose, with phenolics predominantly bound. These bound phenolics are covalently linked to cell wall polysaccharides or lignin networks through ester, ether, or glycosidic bonds. During in vitro fermentation, microbial esterases and glycosidases hydrolyze these bonds stepwise, gradually releasing bioactive free phenolics and oligosaccharides. This sustained release allows J1 residues to continuously provide utilizable carbon sources and phenolic modulators for microbes, thereby supporting efficient SCFA production.

In contrast, in late-ripe (J2/J3) jackfruit, cell wall softening and degradation reduce bound phenolic content and diminish the matrix-mediated slow-release effect, ultimately lowering SCFA yields.

By demonstrating that gut microbiota generate SCFAs via fermentation of matrix-bound phenolics, and that fruit ripeness modulates this process by altering jackfruit cell-wall properties, this study reveals a diet-modifiable mechanistic axis. These microbiota–SCFA shifts are widely associated with improved glycemic and lipid handling and reduced intestinal inflammation in vivo, suggesting potential benefits for metabolic and gut-barrier health. Nevertheless, these implications require validation in human or animal studies.

Furthermore, given the inherent simplification of in vitro models and the substantial inter-individual variability in microbiota composition, the findings should not be directly extrapolated to quantitative dietary recommendations. Consequently, rigorous in vivo dose–response studies are required to establish physiologically effective thresholds.

## 4. Conclusions

This study investigates the changes in polyphenol content, composition, and bioactivity in jackfruit during ripening, in vitro digestion, and colonic fermentation and their effects on gut microbiota and short-chain fatty acid production. Increasing ripeness leads to higher levels of free polyphenols and lower levels of soluble conjugated and insoluble bound polyphenols. Antioxidant activity increases with ripeness, whereas enzyme-inhibitory activity shows an opposite, declining trend. Jackfruit polyphenols undergo dynamic structural and compositional transformations during digestion and colonic fermentation, resulting in sustained accumulation of free polyphenols and progressive depletion of soluble conjugated and insoluble bound forms. Their antioxidant activity decreases during the gastric and intestinal phases but increases initially before declining during colonic fermentation. After digestion, enzyme-inhibitory activity shows two trends: reduced α-glucosidase and xanthine oxidase, and enhanced pancreatic lipase inhibition. Moreover, jackfruit polyphenols selectively promote beneficial bacteria such as *Bifidobacterium* and *Lactobacillus* while suppressing harmful taxa such as *Bacteroides fragilis*. They also enhance the production of short-chain fatty acids, including acetic and butyric acids, which contribute to maintaining gut homeostasis, although these probiotic effects diminish with increasing ripeness. Overall, this study reveals the dual regulatory actions of jackfruit polyphenols on metabolic enzymes and microbiota-SCFA pathways, providing a rationale for functional food development. In particular, the ripeness-dependent polyphenol profile suggests opportunities to design targeted jackfruit-based ingredients with tailored functionalities for glycemic and lipid management as well as gut-health support. From an industrial perspective, the stability of these polyphenols during food processing and storage (e.g., heat treatment and shelf-life conditions) warrants further investigation to facilitate their practical application. In particular, emerging food processing and delivery strategies, such as microencapsulation, co-crystallization, and composite hydrogel systems, have shown promise in protecting polyphenols from degradation during processing and gastrointestinal digestion. These approaches may enhance the stability of polyphenols under thermal and storage conditions, regulate their release behavior during digestion, and ultimately improve their bioaccessibility in the gastrointestinal tract. Incorporating such technologies into jackfruit-based ingredient development could represent a feasible strategy to maximize the functional potential of polyphenols in real food systems.

## Figures and Tables

**Table 1:** Qualitative Profiling (HPLC–HR TOF MS^2^) and Quantitative Analysis (HPLC) of Polyphenols in Jackfruit at Different Maturity Stages and Binding Forms.

Compounds	Molecular Formula	Molecular Weight	RT (min)	Adduct	Mass Error (ppm)	Observed *m*/*z*	J1			J2			J3		
							FP	CP	BP	FP	CP	BP	FP	CP	BP
Gallic acid	C_7_H_6_O_5_	170.1	1.8	[M-H]^−^	1.9	169.01	0.05 ± 0	0.076 ± 0.007	0.04 ± 0.001	0.053 ± 0.001	0.072 ± 0.001	0.044 ± 0	0.043 ± 0.001	0.063 ± 0.001	0.032 ± 0
Chlorogenic Acid	C_16_H_18_O_9_	354.3	3.1	[M-H]^−^	0.3	353.1	0.034 ± 0.003	0.035 ± 0.001	0.029 ± 0.003	0.04 ± 0.001	0.034 ± 0.001	0.032 ± 0.001	0.028 ± 0	0.039 ± 0.001	0.033 ± 0.002
p-Hydroxybenzoic acid	C_7_H_6_O_3_	138.1	2.3	[M+HCOO]^−^	2.9	183	0.04 ± 0.001	0.139 ± 0.001	0.04 ± 0	0.029 ± 0.001	0.146 ± 0.001	0.047 ± 0.001	0.029 ± 0.001	0.086 ± 0	0.05 ± 0
Caffeic acid	C_9_H_8_O_4_	180.2	3.7	[M-H]^−^	0.9	179	0.047 ± 0.002	0.052 ± 0.005	0.031 ± 0.001	0.048 ± 0.001	0.048 ± 0.001	0.036 ± 0.001	0.048 ± 0.001	0.036 ± 0.001	0.031 ± 0
Homovanillic acid	C_9_H_10_O_4_	182.2	4	[M-H]^−^	0.7	181.1	0.062 ± 0	0.067 ± 0.002	0.037 ± 0.001	0.037 ± 0	0.07 ± 0.004	0.034 ± 0.001	0.041 ± 0.002	0.146 ± 0	0.042 ± 0
3-Hydroxyphenylacetic acid	C_8_H_8_O_3_	152.2	4.7	[M-H]^−^	1.9	151	0.045 ± 0	0.069 ± 0.001	0.039 ± 0	ND	0.048 ± 0.001	0.038 ± 0	0.035 ± 0.001	0.047 ± 0	0.060± 0
Sinapic acid	C_11_H_12_O_5_	224.2	4.6	[M-H]^−^	0.5	223.1	0.057 ± 0	0.051 ± 0.002	0.032 ± 0.001	0.034 ± 0.001	0.04 ± 0	0.032 ± 0.001	0.033 ± 0	0.038 ± 0.001	0.032 ± 0
Ferulic acid	C_10_H_10_O_4_	194.2	4.6	[M-H]^−^	0.3	193.1	0.029 ± 0	0.025 ± 0.004	0.022 ± 0.003	0.024 ± 0.001	0.025 ± 0.001	0.025 ± 0	0.022 ± 0.001	0.025 ± 0.001	0.025 ± 0
2-Methoxycinnamaldehyde	C_10_H_10_O_2_	162.2	5.5	[M+NH_4_]^+^	2.4	325.3	0.031 ± 0.001	0.027 ± 0.005	0.026 ± 0.004	0.029 ± 0.001	0.028 ± 0.002	0.028 ± 0.001	0.029 ± 0.002	0.028 ± 0.002	0.028 ± 0
(+)-Catechin	C_15_H_14_O_6_	290.3	3.3	[M-H]^−^	2.3	289.1	0.046 ± 0.002	0.057 ± 0.001	0.038 ± 0.001	0.089 ± 0.001	0.053 ± 0.002	0.034 ± 0	0.088 ± 0	0.046 ± 0.001	0.042 ± 0
vitexin-2″-o-rhamnoside	C_27_H_30_O_14_	578.5	4.2	[M-H]^−^	4.1	577.2	0.073 ± 0.003	0.032 ± 0.005	0.029 ± 0.002	0.052 ± 0.001	0.035 ± 0.001	0.031 ± 0.001	0.052 ± 0.001	0.068 ± 0.001	0.042 ± 0.001
Hyperoside	C_21_H_20_O_12_	464.4	4.5	[M-H]^−^	5.9	463.1	0.069 ± 0.001	0.029 ± 0.001	ND	0.030 ± 0.001	0.03 ± 0.002	0.029 ± 0	0.029 ± 0	0.032 ± 0.001	0.028 ± 0.002
(−)-Epicatechin gallate	C_22_H_18_O_10_	442.4	4.9	[M-H]^−^	0.2	441.1	0.041 ± 0	0.038 ± 0.004	0.034 ± 0.001	0.035 ± 0	0.036 ± 0	0.035 ± 0.001	0.035 ± 0	0.036 ± 0.001	0.034 ± 0
Taxifolin	C_15_H_12_O_7_	304.3	4.7	[M-H]^−^	0.7	303.1	0.033 ± 0.001	ND	0.033 ± 0.001	0.033 ± 0	0.034 ± 0	0.034 ± 0	0.033 ± 0.001	0.033 ± 0.002	ND
Glycitein	C_16_H_12_O_5_	284.3	5.3	[M-H]^−^	1.5	283.1	0.027 ± 0.003	0.026 ± 0.001	ND	0.026 ± 0.003	0.026 ± 0	ND	ND	0.028 ± 0.001	0.028 ± 0.001
Quercetin	C_15_H_10_O_7_	302.2	5.4	[M+CH_3_COO]^−^	3.3	361.1	0.031 ± 0	0.032 ± 0	0.028 ± 0.001	0.030 ± 0.001	0.03 ± 0.001	0.028 ± 0	0.030 ± 0.001	0.029 ± 0.002	0.028 ± 0.002
Naringenin	C_15_H_12_O_5_	272.3	4.3	[M+HCOO]^−^	0.3	317.1	0.03 ± 0.001	0.032 ± 0.001	ND	ND	0.031 ± 0	ND	ND	0.031 ± 0.001	ND
Mulberrin	C_25_H_26_O_6_	422.5	3.7	[M+NH_4_]^+^	4.6	440.2	0.054 ± 0	0.040 ± 0.001	ND	0.039 ± 0.001	0.056 ± 0.001	ND	0.036 ± 0	0.054 ± 0.001	0.033 ± 0

Values are expressed as mean ± standard deviation. The results represent the highest quantified polyphenol levels observed in each sample across the CG, GP, and IP stages, as well as the CF stage (0.3, 2, 4, 8, 16, and 24 h). ND indicates that the compound was not detected. Complete quantitative data are provided in Tables S2–S4.

## Data Availability

The original contributions presented in this study are included in the article/Supplementary Materials. Further inquiries can be directed to the corresponding author.

## Supplementary Materials

The following supporting information can be downloaded at: https://www.mdpi.com/article/10.3390/antiox15010026/s1, Figure S1: Different maturity of jackfruit. From left to right are J1 (unripe), J2 (underripe), J3 (ripe); Table S1: Per-sample sequencing read counts and quality metrics before and after quality filtering for 16S rRNA amplicon data; Table S2: HPLC quantitative analysis of different forms of polyphenols in jackfruit (J1) during in vitro digestion and colon fermentation; Table S3: HPLC quantitative analysis of different forms of polyphenols in jackfruit (J2) during in vitro digestion and colon fermentation; Table S4: HPLC quantitative analysis of different forms of polyphenols in jackfruit (J3) during in vitro digestion and colon fermentation; Table S5: Analysis of α-diversity of intestinal microbial community during in vitro colonic fermentation of jackfruit with different maturity; Table S6: Determination of SCFAs content in different maturity jackfruit during in vitro colonic fermentation. Abbreviation: Control, fresh slurry control.
