# Supplementary material for: Dynamic Changes in Jackfruit (Artocarpus heterophyllus Lam.) Polyphenols During Maturation and In Vitro Digestion and Their Effects on the Gut Microbiota
Source: Antioxidants (Basel). 2025 Dec 23;15(1):26. doi: 10.3390/antiox15010026 (PMC12837454; doi:10.3390/antiox15010026)
Supplement: Supplementary file 1 [file antioxidants-15-00026-s001.zip › antioxidants-4002730-supplementary.docx]

Fig. S1 Different maturity of jackfruit. From left to right are J1 ( unripe ), J2 ( underripe ), J3 ( ripe ).

Table S1. Per-sample sequencing read counts and quality metrics before and after quality filtering for 16S rRNA amplicon data.

| **Sample** | **Raw_tags** | **Clean_tags** | **Effective_tags** | **Effective_ratio(%)** | **Effective_bases(nt)** | **Min_length** | **Max_length** | **Mean_length** | **Q20(%)** | **Q30(%)** | **GC(%)** |
| --- | --- | --- | --- | --- | --- | --- | --- | --- | --- | --- | --- |
| Control-1 | 103,413 | 102,322 | 83,285 | 80.54 | 34,742,260 | 338 | 430 | 417 | 98.1 | 94.08 | 52.67 |
| Control-2 | 103,558 | 102,392 | 79,193 | 76.47 | 33,111,781 | 245 | 431 | 418 | 97.89 | 93.58 | 53.09 |
| Control-3 | 105,197 | 104,157 | 88,054 | 83.7 | 36,604,210 | 318 | 438 | 416 | 98.26 | 94.51 | 52.78 |
| J1-CF-0.3h-1 | 102,981 | 101,875 | 89,290 | 86.71 | 37,526,625 | 365 | 433 | 420 | 98.06 | 94.02 | 51.66 |
| J1-CF-0.3h-2 | 92,879 | 91,904 | 83,139 | 89.51 | 34,975,920 | 262 | 430 | 421 | 98.03 | 93.99 | 51.84 |
| J1-CF-0.3h-3 | 102,224 | 101,092 | 92,379 | 90.37 | 38,873,583 | 330 | 431 | 421 | 98.01 | 93.93 | 51.83 |
| J2-CF-0.3h-1 | 106,265 | 105,077 | 91,895 | 86.48 | 38,539,284 | 261 | 437 | 419 | 98 | 93.92 | 51.85 |
| J2-CF-0.3h-2 | 106,122 | 105,035 | 88,634 | 83.52 | 37,410,919 | 343 | 432 | 422 | 98.14 | 94.25 | 52.4 |
| J2-CF-0.3h-3 | 84,942 | 84,038 | 74,775 | 88.03 | 31,482,725 | 399 | 438 | 421 | 98.03 | 93.97 | 51.94 |
| J3-CF-0.3h-1 | 81,486 | 80,553 | 73,196 | 89.83 | 30,808,009 | 259 | 437 | 421 | 98.03 | 94.01 | 51.87 |
| J3-CF-0.3h-2 | 65,331 | 64,660 | 57,380 | 87.83 | 24,154,815 | 340 | 430 | 421 | 98.15 | 94.27 | 51.75 |
| J3-CF-0.3h-3 | 105,355 | 104,177 | 95,399 | 90.55 | 40,137,752 | 301 | 430 | 421 | 98.08 | 94.08 | 51.79 |
| J1-CF-2h-1 | 104,850 | 103,427 | 90,461 | 86.28 | 38,096,214 | 252 | 436 | 421 | 97.87 | 93.59 | 51.87 |
| J1-CF-2h-2 | 104,867 | 103,727 | 90,891 | 86.67 | 38,064,123 | 261 | 439 | 419 | 98.09 | 94.14 | 51.54 |
| J1-CF-2h-3 | 109,694 | 108,686 | 96,259 | 87.75 | 40,407,349 | 271 | 432 | 420 | 98.03 | 94.13 | 51.8 |
| J2-CF-2h-1 | 106,311 | 105,097 | 98,820 | 92.95 | 41,652,967 | 262 | 446 | 422 | 98.25 | 94.49 | 51.78 |
| J2-CF-2h-2 | 103,091 | 101,984 | 95,227 | 92.37 | 40,239,041 | 314 | 438 | 423 | 98.15 | 94.28 | 51.77 |
| J2-CF-2h-3 | 103,855 | 102,647 | 96,383 | 92.81 | 40,705,345 | 317 | 438 | 422 | 98.09 | 94.16 | 51.76 |
| J3-CF-2h-1 | 104,405 | 103,243 | 94,547 | 90.56 | 39,797,655 | 315 | 430 | 421 | 98.16 | 94.28 | 51.9 |
| J3-CF-2h-2 | 107,489 | 106,391 | 100,414 | 93.42 | 42,345,680 | 321 | 430 | 422 | 98.18 | 94.38 | 51.77 |
| J3-CF-2h-3 | 102,078 | 101,015 | 95,360 | 93.42 | 40,196,582 | 365 | 431 | 422 | 98.25 | 94.53 | 51.75 |
| J1-CF-4h-1 | 106,479 | 104,889 | 98,961 | 92.94 | 42,026,941 | 365 | 446 | 425 | 98.09 | 94.17 | 52.86 |
| J1-CF-4h-2 | 105,877 | 104,787 | 97,101 | 91.71 | 41,231,427 | 310 | 430 | 425 | 98.23 | 94.51 | 52.6 |
| J1-CF-4h-3 | 79,808 | 79,034 | 75,246 | 94.28 | 31,950,762 | 388 | 430 | 425 | 98.22 | 94.47 | 52.69 |
| J2-CF-4h-1 | 103,441 | 102,363 | 95,053 | 91.89 | 40,428,206 | 203 | 431 | 425 | 98.13 | 94.13 | 52.82 |
| J2-CF-4h-2 | 89,965 | 89,149 | 84,534 | 93.96 | 35,935,997 | 338 | 430 | 425 | 98.26 | 94.52 | 52.31 |
| J2-CF-4h-3 | 116,611 | 115,560 | 105,478 | 90.45 | 44,837,321 | 365 | 436 | 425 | 98.18 | 94.33 | 53.06 |
| J3-CF-4h-1 | 104,028 | 102,912 | 90,240 | 86.75 | 38,215,797 | 317 | 442 | 424 | 98.23 | 94.43 | 52.88 |
| J3-CF-4h-2 | 106,753 | 105,616 | 101,145 | 94.75 | 42,777,576 | 262 | 430 | 423 | 98.12 | 94.17 | 52.56 |
| J3-CF-4h-3 | 106,436 | 105,132 | 98,750 | 92.78 | 41,802,047 | 310 | 438 | 423 | 98.06 | 94.04 | 52.07 |
| J1-CF-8h-1 | 103,086 | 102,070 | 96,440 | 93.55 | 41,054,180 | 286 | 438 | 426 | 98.17 | 94.36 | 53.64 |
| J1-CF-8h-2 | 102,327 | 101,315 | 96,346 | 94.16 | 41,007,722 | 338 | 431 | 426 | 98.17 | 94.35 | 53.48 |
| J1-CF-8h-3 | 106,636 | 105,525 | 97,028 | 90.99 | 41,280,893 | 318 | 430 | 426 | 98.23 | 94.37 | 53.74 |
| J2-CF-8h-1 | 75,854 | 75,219 | 70,579 | 93.05 | 30,147,142 | 297 | 431 | 427 | 98.01 | 93.94 | 54.31 |
| J2-CF-8h-2 | 62,988 | 62,356 | 58,248 | 92.47 | 24,788,463 | 265 | 430 | 426 | 98.23 | 94.49 | 53.82 |
| J2-CF-8h-3 | 105,510 | 104,496 | 95,684 | 90.69 | 40,608,356 | 365 | 435 | 424 | 98.27 | 94.54 | 54.38 |
| J3-CF-8h-1 | 89,509 | 88,640 | 83,085 | 92.82 | 35,373,192 | 261 | 431 | 426 | 98.19 | 94.3 | 53.74 |
| J3-CF-8h-2 | 102,127 | 101,190 | 95,537 | 93.55 | 40,660,687 | 364 | 430 | 426 | 98.23 | 94.45 | 53.72 |
| J3-CF-8h-3 | 94,163 | 93,280 | 84,927 | 90.19 | 36,165,564 | 262 | 440 | 426 | 98.28 | 94.55 | 54.01 |
| J1-CF-16h-1 | 102,214 | 100,864 | 91,658 | 89.67 | 38,967,340 | 375 | 442 | 425 | 98.03 | 94.09 | 54.3 |
| J1-CF-16h-2 | 82,953 | 82,266 | 78,373 | 94.48 | 33,258,473 | 403 | 430 | 424 | 98.27 | 94.55 | 54.54 |
| J1-CF-16h-3 | 105,068 | 104,054 | 99,187 | 94.4 | 42,146,088 | 265 | 438 | 425 | 98.31 | 94.69 | 54.87 |
| J2-CF-16h-1 | 103,254 | 102,194 | 94,051 | 91.09 | 39,871,995 | 389 | 438 | 424 | 98.35 | 94.8 | 54.97 |
| J2-CF-16h-2 | 112,982 | 111,952 | 97,378 | 86.19 | 41,269,639 | 375 | 440 | 424 | 98.39 | 94.73 | 55.9 |
| J2-CF-16h-3 | 102,866 | 101,749 | 91,471 | 88.92 | 38,830,513 | 338 | 446 | 424 | 98.32 | 94.74 | 54.68 |
| J3-CF-16h-1 | 105,890 | 104,964 | 93,260 | 88.07 | 39,524,945 | 313 | 438 | 424 | 98.19 | 94.24 | 55.96 |
| J3-CF-16h-2 | 102,152 | 101,228 | 92,351 | 90.41 | 39,148,748 | 334 | 438 | 424 | 98.26 | 94.6 | 55.99 |
| J3-CF-16h-3 | 83,159 | 82,623 | 78,072 | 93.88 | 33,228,042 | 270 | 442 | 426 | 98.19 | 94.4 | 55.28 |
| J1-CF-24h-1 | 108,755 | 107,786 | 102,167 | 93.94 | 43,124,190 | 362 | 438 | 422 | 98.3 | 94.72 | 55.71 |
| J1-CF-24h-2 | 113,727 | 112,581 | 106,599 | 93.73 | 45,032,327 | 319 | 438 | 422 | 98.14 | 94.31 | 55.7 |
| J1-CF-24h-3 | 106,202 | 105,307 | 97,995 | 92.27 | 41,451,874 | 389 | 438 | 423 | 98.31 | 94.72 | 55.44 |
| J2-CF-24h-1 | 103,145 | 102,152 | 94,041 | 91.17 | 39,695,162 | 261 | 438 | 422 | 98.15 | 94.37 | 55.51 |
| J2-CF-24h-2 | 103,734 | 102,695 | 95,362 | 91.93 | 40,349,039 | 265 | 440 | 423 | 98.22 | 94.47 | 54.65 |
| J2-CF-24h-3 | 62,223 | 61,647 | 58,792 | 94.49 | 24,872,401 | 375 | 430 | 423 | 98.25 | 94.58 | 54.76 |
| J3-CF-24h-1 | 105,709 | 104,877 | 99,960 | 94.56 | 42,548,211 | 236 | 440 | 426 | 98.26 | 94.47 | 55.73 |
| J3-CF-24h-2 | 102,934 | 102,017 | 94,478 | 91.79 | 40,205,997 | 373 | 431 | 426 | 98.09 | 94.12 | 55.78 |
| J3-CF-24h-3 | 86,806 | 86,009 | 81,693 | 94.11 | 34,834,965 | 328 | 438 | 426 | 98.33 | 94.56 | 55.69 |

Raw Tags are the original sequencing reads. Clean Tags are reads retained after removing low-quality and short sequences. Effective Tags are reads remaining after chimera removal and were used for downstream analyses. Effective Ratio (%) = (Effective Tags / Raw Tags) × 100. Effective Bases (nt) indicates the total number of bases in Effective Tags. Min/Max/Mean Length denote the minimum, maximum, and mean read length of Effective Tags, respectively. Q20 (%) and Q30 (%) indicate the percentage of bases in Clean Tags with Phred scores ≥20 (error rate <1%) and ≥30 (error rate <0.1%), respectively. GC (%) indicates the GC content of Clean Tags.

Table S2. HPLC quantitative analysis of different forms of polyphenols in jackfruit ( J1 ) during *in vitro* digestion and colon fermentation.

| **Compounds** | **Gallic acid** | **Chlorogenic Acid** | **p-Hydroxybenzoic acid** | **Caffeic acid** | **Homovanillic acid** | **3-Hydroxyphenylacetic acid** | **Sinapic acid** | **Ferulic acid** | **2-Methoxycinnamaldehyde** | **(+)-Cianidanol** | **vitexin-2″-o-rhamnoside** | **Hyperoside** | **(-)-Epicatechin gallate** | **Taxifolin** | **Glycitein** | **Quercetin** | **Naringenin** | **Mulberrin** |
| --- | --- | --- | --- | --- | --- | --- | --- | --- | --- | --- | --- | --- | --- | --- | --- | --- | --- | --- |
| J1-CG-FP | 0.031 ± 0.001^ABgh^ | 0.030 ± 0.002^BCde^ | ND | 0.031 ± 0.001^ABc^ | ND | 0.033 ± 0.001^ABe^ | ND | 0.025 ± 0.003^Db^ | ND | 0.034 ± 0.001^Ahi^ | ND | 0.031 ± 0.002^BCb^ | ND | ND | 0.027 ± 0.003^CDa^ | ND | ND | ND |
| J1-CG-CP | 0.033 ± 0.002^BCgh^ | 0.030 ± 0^BCde^ | 0.029 ± 0.001^CDi^ | 0.033 ± 0.005^BCc^ | 0.033 ± 0.001^BCh^ | 0.035 ± 0.002^Be^ | ND | 0.025 ± 0.004^Db^ | ND | 0.041 ± 0.003^Acd^ | ND | ND | ND | ND | ND | ND | 0.031 ± 0.004^BCa^ | 0.040 ± 0.001^Ab^ |
| J1-CG-BP | 0.029 ± 0^Aij^ | ND | ND | ND | ND | ND | ND | ND | ND | ND | 0.029 ± 0.001^Ade^ | ND | ND | ND | ND | ND | ND | ND |
| J1-GP-FP | 0.05 ± 0^Ade^ | ND | ND | 0.03 ± 0.005^BCc^ | 0.031 ± 0.002^BCa^ | 0.032 ± 0.001^BCe^ | 0.032 ± 0.001^BCg^ | 0.023 ± 0.001^Ebc^ | 0.027 ± 0^DEb^ | 0.033 ± 0.001^Bhi^ | 0.028 ± 0.002^DEde^ | 0.029 ± 0.003^CDb^ | ND | ND | 0.026 ± 0.001^Ea^ | ND | ND | ND |
| J1-GP-CP | 0.056 ± 0.002^Bbc^ | 0.029 ± 0^Ede^ | 0.065 ± 0^Ad^ | 0.046 ± 0.001^Cb^ | 0.066 ± 0^Aa^ | 0.055 ± 0.001^Bb^ | 0.036 ± 0.001^Def^ | 0.022 ± 0.003^Gbc^ | 0.027 ± 0.005^Eb^ | 0.042 ± 0.004^Cc^ | 0.029 ± 0.001^Ede^ | 0.029 ± 0.002^Eb^ | 0.034 ± 0.004^Dbc^ | ND | 0.026 ± 0.004^EFa^ | 0.029 ± 0^Eb^ | ND | 0.036 ± 0^Dc^ |
| J1-GP-BP | 0.028 ± 0.002^Aij^ | ND | 0.027 ± 0.00^2Ai^ | ND | ND | ND | ND | ND | ND | ND | ND | ND | ND | ND | ND | ND | ND | ND |
| J1-IP-FP | 0.029 ± 0.003^Ahi^ | ND | ND | ND | ND | ND | 0.031 ± 0.002^Ag^ | ND | ND | ND | ND | ND | ND | ND | ND | ND | ND | ND |
| J1-IP-CP | 0.076 ± 0.007^Aa^ | 0.03 ± 0.003^EFcd^ | 0.077 ± 0.001^Ac^ | 0.052 ± 0.005^Ca^ | 0.067 ± 0.002^Ba^ | 0.069 ± 0.001^Ba^ | 0.036 ± 0^DEef^ | 0.023 ± 0^Gbc^ | 0.027 ± 0.001^FGb^ | 0.049 ± 0.002^Cb^ | 0.03 ± 0^EFde^ | 0.029 ± 0.001^EFb^ | 0.033 ± 0.003^Ec^ | ND | 0.026 ± 0.001^FGa^ | 0.032 ± 0^Ea^ | ND | 0.039 ± 0^Db^ |
| J1-IP-BP | 0.028 ± 0.002^Aij^ | ND | ND | ND | ND | ND | ND | ND | ND | ND | ND | ND | ND | ND | ND | ND | ND | ND |
| J1-CF-0.3h-FP | 0.027 ± 0.001^Ej^ | 0.034 ± 0.003^Dab^ | 0.04 ± 0.001^Cg^ | ND | 0.055 ± 0.002^Bc^ | ND | 0.057 ± 0^Ba^ | ND | 0.031 ± 0.001^DEa^ | 0.038 ± 0.003^Cde^ | 0.069 ± 0.006^Aab^ | 0.069 ± 0.001^Aa^ | 0.041 ± 0^Ca^ | ND | 0.024 ± 0.003^Ea^ | 0.031 ± 0^DEa^ | 0.03 ± 0.001^DEa^ | 0.054 ± 0^Ba^ |
| J1-CF-0.3h-CP | 0.053 ± 0.001^Acd^ | 0.033 ± 0.002^Dab^ | 0.043 ± 0.005^Cf^ | 0.032 ± 0.004^Dc^ | 0.048 ± 0^Bd^ | 0.032 ± 0^De^ | 0.033 ± 0.004^Dfg^ | 0.02 ± 0.002^Fc^ | 0.026 ± 0.001^Eb^ | 0.057 ± 0.001^Aa^ | 0.032 ± 0.005^Dd^ | ND | 0.035 ± 0.001^Dbc^ | ND | ND | ND | 0.03 ± 0D^Ea^ | ND |
| J1-CF-0.3h-BP | 0.04 ± 0.001^Af^ | 0.029 ± 0.003^Cde^ | 0.04 ± 0^Ag^ | 0.031 ± 0.001^BCc^ | 0.034 ± 0.005^Bgh^ | 0.034 ± 0^Be^ | 0.032 ± 0^BCg^ | 0.02 ± 0.001^Dc^ | 0.026 ± 0.005^Cb^ | 0.038 ± 0.001^ABde^ | 0.029 ± 0.002^Cde^ | ND | 0.034 ± 0.001^Bbc^ | ND | ND | ND | ND | ND |
| J1-CF-2h-FP | 0.03 ± 0.001^BCgh^ | 0.033 ± 0.002^BCab^ | ND | ND | 0.059 ± 0.004^Ab^ | ND | ND | 0.029 ± 0^Ca^ | 0.026 ± 0.001^Db^ | 0.031 ± 0.005^BCij^ | 0.027 ± 0^Cde^ | ND | 0.034 ± 0.001^Bbc^ | ND | ND | 0.028 ± 0.001^Cb^ | ND | 0.034 ± 0.001^Bde^ |
| J1-CF-2h-CP | 0.057 ± 0.002^Ab^ | 0.035 ± 0.001^CDa^ | 0.061 ± 0^Ac^ | 0.031 ± 0^DEc^ | 0.042 ± 0.002^Bc^ | 0.037 ± 0.005^Cd^ | 0.036 ± 0.001^Cef^ | 0.02 ± 0.001^Gc^ | 0.026 ± 0^Fb^ | 0.037 ± 0.002^Cef^ | 0.028 ± 0.005^EFde^ | ND | 0.038 ± 0.004^BCab^ | ND | ND | ND | 0.03 ± 0.001^EFa^ | ND |
| J1-CF-2h-BP | 0.033 ± 0.001^BCgh^ | 0.028 ± 0.002^De^ | 0.038 ± 0^Ag^ | 0.031 ± 0^Cc^ | ND | 0.033 ± 0.001^BCe^ | 0.032 ± 0.001^BCg^ | 0.02 ± 0.001^Ec^ | 0.026 ± 0.002^Db^ | 0.034 ± 0.001^Bgh^ | 0.027 ± 0^Dde^ | ND | ND | ND | ND | ND | ND | ND |
| J1-CF-4h-FP | 0.031 ± 0.003^Egh^ | 0.034 ± 0^Dab^ | ND | ND | 0.059 ± 0.001^Bb^ | ND | ND | 0.021 ± 0.002^Gbc^ | 0.026 ± 0.001^Fb^ | 0.042 ± 0.002^Cc^ | 0.065 ± 0.002^Ab^ | ND | ND | 0.033 ± 0.001^DEa^ | ND | 0.028 ± 0^Fb^ | ND | ND |
| J1-CF-4h-CP | 0.051 ± 0^Ad^ | 0.028 ± 0^Ee^ | 0.029 ± 0^Ei^ | 0.032 ± 0.001^Dc^ | 0.037 ± 0.003^BCfg^ | 0.032 ± 0^De^ | 0.039 ± 0.002^Bde^ | 0.02 ± 0.001^Fc^ | ND | 0.029 ± 0.001^Ek^ | 0.027 ± 0^Ede^ | ND | 0.035 ± 0.002^Cbc^ | ND | ND | ND | 0.032 ± 0.001^Da^ | ND |
| J1-CF-4h-BP | 0.031 ± 0.001^CDgh^ | ND | 0.04 ± 0^Ag^ | 0.03 ± 0^Dc^ | 0.031 ± 0.001^CDh^ | ND | 0.032 ± 0.001^CDg^ | 0.022 ± 0.003^Fbc^ | 0.026 ± 0.004^Eb^ | 0.036 ± 0^Bfg^ | ND | ND | 0.034 ± 0.001^BCbc^ | ND | ND | ND | ND | ND |
| J1-CF-8h-FP | 0.031 ± 0.001^Egh^ | 0.034 ± 0.002^Dab^ | ND | ND | 0.062 ± 0^Bb^ | 0.045 ± 0^Cc^ | ND | 0.021 ± 0.002^Gbc^ | 0.026 ± 0.001^Fb^ | 0.046 ± 0.002^Cb^ | 0.073 ± 0.003^Aa^ | ND | ND | 0.033 ± 0^DEa^ | ND | 0.028 ± 0.001^Fb^ | ND | 0.033 ± 0^DEe^ |
| J1-CF-8h-CP | 0.054 ± 0.001^Bbc^ | 0.031 ± 0^GHbc^ | 0.139 ± 0.001^Aa^ | 0.045 ± 0.002^Db^ | ND | 0.033 ± 0.001^FGe^ | 0.051 ± 0.002^Cb^ | 0.021 ± 0.002^Jbc^ | ND | 0.03 ± 0^HIjk^ | 0.032 ± 0.001^GHd^ | 0.028 ± 0.00^1Ib^ | 0.038 ± 0^Eab^ | ND | ND | ND | 0.031 ± 0.002^GHa^ | 0.035 ± 0^Fcd^ |
| J1-CF-8h-BP | 0.034 ± 0.001^BCg^ | ND | 0.04 ± 0.002^Ag^ | 0.03 ± 0.001^DEc^ | ND | 0.039 ± 0^Ad^ | 0.032 ± 0.002^CDg^ | 0.022 ± 0.001^Gbc^ | 0.026 ± 0^Fb^ | 0.036 ± 0^Bfg^ | 0.026 ± 0.001^Fe^ | ND | 0.034 ± 0.002^BCbc^ | 0.033 ± 0.001^Ca^ | ND | 0.028 ± 0.001^EFb^ | ND | ND |
| J1-CF-16h-FP | 0.032 ± 0^Egh^ | 0.032 ± 0.001^Eab^ | ND | 0.047 ± 0.002^Bab^ | 0.038 ± 0.002^Df^ | ND | ND | 0.021 ± 0^Hbc^ | 0.027 ± 0^Fb^ | 0.041 ± 0.001^Ccd^ | 0.067 ± 0.002^Ab^ | 0.028 ± 0.001^Fb^ | 0.034 ± 0.004^Ebc^ | 0.033 ± 0^Ea^ | 0.024 ± 0^Ga^ | 0.028 ± 0.001^Fb^ | ND | ND |
| J1-CF-16h-CP | 0.047 ± 0.001^Be^ | 0.03 ± 0.002^Ecd^ | 0.133 ± 0.001^Ab^ | 0.047 ± 0.002^Bab^ | ND | ND | 0.04 ± 0.003^Cd^ | 0.02 ± 0.001^Fc^ | ND | 0.029 ± 0.002^Ek^ | 0.028 ± 0^Ede^ | ND | 0.034 ± 0^Dbc^ | ND | ND | ND | 0.03 ± 0^Ea^ | ND |
| J1-CF-16h-BP | 0.027 ± 0.002^Fj^ | ND | 0.039 ± 0^Ag^ | 0.03 ± 0.001^Ec^ | 0.037 ± 0.001^ABfg^ | ND | 0.032 ± 0^DEg^ | 0.022 ± 0.001^Gbc^ | 0.026 ± 0.002^Fb^ | 0.036 ± 0.001^BCfg^ | 0.027 ± 0^Fde^ | ND | 0.034 ± 0^CDbc^ | 0.033 ± 0.002^Da^ | ND | ND | ND | ND |
| J1-CF-24h-FP | 0.032 ± 0.002^Cgh^ | 0.032 ± 0.001^Cab^ | ND | 0.047 ± 0^Aab^ | 0.038 ± 0.001^Bf^ | ND | ND | 0.02 ± 0.001^Ec^ | 0.026 ± 0^Db^ | 0.04 ± 0.002^Bcd^ | 0.039 ± 0^Bc^ | 0.028 ± 0.004^Db^ | 0.034 ± 0^Cbc^ | 0.033 ± 0.002^Ca^ | ND | 0.028 ± 0^Db^ | ND | 0.033 ± 0.001^Ce^ |
| J1-CF-24h-CP | 0.05 ± 0.005^Bde^ | 0.03 ± 0.001^DEcd^ | 0.133 ± 0.001^Ab^ | 0.047 ± 0.006^BCab^ | ND | ND | 0.044 ± 0^Cc^ | 0.02 ± 0.001^Fc^ | 0.026 ± 0^Eb^ | 0.03 ± 0.001^DEjk^ | 0.026 ± 0.005^Ee^ | ND | 0.034 ± 0.001^Dbc^ | ND | ND | ND | 0.03 ± 0^DEa^ | ND |
| J1-CF-24h-BP | 0.04 ± 0.002^Af^ | ND | 0.033 ± 0^Ch^ | 0.03 ± 0.001^Dc^ | 0.032 ± 0^Ch^ | ND | ND | 0.02 ± 0^Gc^ | 0.026 ± 0.002^Fb^ | 0.035 ± 0^Bfg^ | 0.028 ± 0.001^Ede^ | ND | ND | ND | ND | 0.028 ± 0.001^Eb^ | ND | ND |

ND means not detected. The values in each grid represent the mean ± standard deviation. The different uppercase letters in the same row represent significant differences, and the different lowercase letters in the same column represent significant differences ( p < 0.05 ).

Table S3. HPLC quantitative analysis of different forms of polyphenols in jackfruit ( J2 ) during *in vitro* digestion and colon fermentation.

| **Compounds** | **Gallic acid** | **Chlorogenic Acid** | **p-Hydroxybenzoic acid** | **Caffeic acid** | **Homovanillic acid** | **3-Hydroxyphenylacetic acid** | **Sinapic acid** | **Ferulic acid** | **2-Methoxycinnamaldehyde** | **(+)-Cianidanol** | **vitexin-2″-o-rhamnoside** | **Hyperoside** | **(-)-Epicatechin gallate** | **Taxifolin** | **Glycitein** | **Quercetin** | **Naringenin** | **Mulberrin** |
| --- | --- | --- | --- | --- | --- | --- | --- | --- | --- | --- | --- | --- | --- | --- | --- | --- | --- | --- |
| J2-CG-FP | 0.032 ± 0.002^ABhi^ | 0.030 ± 0^Bef^ | ND | 0.031 ± 0.001^Bh^ | 0.032 ± 0.002^ABh^ | ND | 0.031 ± 0.001^Bf^ | ND | ND | 0.034 ± 0.001^Aij^ | ND | 0.030 ± 0.001^Ba^ | ND | ND | ND | 0.030 ± 0.001^Ba^ | ND | ND |
| J2-CG-CP | 0.035 ± 0^Cg^ | 0.030 ± 0^Fef^ | 0.030 ± 0.001^Fg^ | 0.033 ± 0^Dfg^ | 0.032 ± 0^DEh^ | 0.035 ± 0.001^Cbc^ | ND | 0.025 ± 0.001^Ha^ | 0.028 ± 0.002^Ga^ | 0.037 ± 0^Bh^ | 0.035 ± 0.001^Cde^ | 0.030 ± 0.001^Fa^ | ND | ND | ND | ND | 0.031 ± 0^EFa^ | 0.056 ± 0.001^Aa^ |
| J2-CG-BP | 0.034 ± 0.001^Agh^ | ND | 0.033 ± 0.003^ABi^ | ND | ND | ND | ND | 0.025 ± 0^Ca^ | 0.028 ± 0.001^Ca^ | 0.033 ± 0.002^ABjk^ | 0.029 ± 0.004^BCfg^ | ND | ND | ND | ND | ND | ND | ND |
| J2-GP-FP | 0.053 ± 0.001^Ab^ | 0.028 ± 0^DEf^ | 0.028 ± 0^DEg^ | 0.03 ± 0.002^CDh^ | 0.031 ± 0^BCh^ | ND | 0.032 ± 0.001^BCde^ | 0.023 ± 0^Gab^ | 0.027 ± 0.001^EFa^ | 0.033 ± 0^Bij^ | 0.030 ± 0^DEfg^ | 0.029 ± 0.001^DEa^ | ND | ND | 0.026 ± 0.003^Fa^ | ND | ND | ND |
| J2-GP-CP | 0.05 ± 0^Cc^ | 0.029 ± 0^Fcf^ | 0.056 ± 0.002^Bf^ | 0.037 ± 0.001^DEd^ | 0.067 ± 0^Ab^ | 0.047 ± 0.005^Ca^ | 0.035 ± 0.001^Ed^ | 0.023 ± 0^Gab^ | 0.027 ± 0.001^FGa^ | 0.04 ± 0^Dg^ | 0.029 ± 0.005^Ffg^ | 0.029 ± 0.001^Fa^ | 0.034 ± 0.004^Ea^ | ND | ND | 0.029 ± 0.005^Fa^ | ND | 0.048 ± 0.001^Cb^ |
| J2-GP-BP | 0.029 ± 0^Ajk^ | ND | ND | ND | ND | ND | ND | ND | ND | 0.03 ± 0.001^Al^ | ND | ND | ND | ND | ND | ND | ND | ND |
| J2-IP-FP | 0.03 ± 0.006^Aij^ | ND | 0.029 ± 0.001^Ag^ | ND | ND | ND | 0.033 ± 0^Ade^ | 0.023 ± 0.001^Bab^ | ND | 0.03 ± 0.002^Al^ | ND | ND | ND | ND | ND | ND | ND | ND |
| J2-IP-CP | 0.072 ± 0.001^Aa^ | 0.032 ± 0.001^EFcd^ | 0.07 ± 0^Ae^ | 0.041 ± 0.001^Cc^ | 0.07 ± 0.004^Aa^ | 0.048 ± 0.001^Ba^ | 0.035 ± 0^Dd^ | 0.023 ± 0.001^Hab^ | 0.027 ± 0^Ga^ | 0.042 ± 0^Cf^ | 0.03 ± 0^Ffg^ | 0.03 ± 0.002^Fa^ | 0.033 ± 0.001^DEa^ | ND | 0.026 ± 0^Ga^ | 0.03 ± 0.001^Fa^ | ND | 0.047 ± 0.001^Bb^ |
| J2-IP-BP | 0.029 ± 0.001^Bjk^ | 0.029 ± 0.002^Bef^ | 0.029 ± 0^Bg^ | ND | ND | 0.032 ± 0.001^Ac^ | 0.031 ± 0.001^ABef^ | ND | ND | 0.03 ± 0^ABl^ | 0.031 ± 0.001^ABef^ | ND | ND | ND | ND | ND | ND | ND |
| J2-CF-0.3h-FP | 0.029 ± 0^Cjk^ | ND | ND | 0.034 ± 0.002^Bef^ | 0.032 ± 0^Bgh^ | ND | 0.034 ± 0.001^Bde^ | 0.02 ± 0^Ed^ | 0.026 ± 0.001^Da^ | 0.054 ± 0^Ae^ | 0.029 ± 0.001^Cfg^ | ND | 0.034 ± 0.001^Ba^ | 0.033 ± 0.002^Ba^ | ND | ND | ND | ND |
| J2-CF-0.3h-CP | 0.047 ± 0^Bd^ | 0.033 ± 0.001^FGbc^ | 0.042 ± 0.001^Dh^ | 0.032 ± 0^Gfg^ | 0.045 ± 0.001^Cc^ | ND | 0.032 ± 0.001^Gde^ | 0.02 ± 0.001^Gd^ | 0.026 ± 0.001^Ia^ | 0.053 ± 0.002^Ae^ | ND | ND | 0.036 ± 0^Ea^ | 0.034 ± 0^Fa^ | ND | ND | 0.03 ± 0.001^Ha^ | 0.034 ± 0^Ffg^ |
| J2-CF-0.3h-BP | 0.044 ± 0^Be^ | 0.032 ± 0.001^EFcd^ | 0.047 ± 0.001^Ag^ | 0.031 ± 0^Fgh^ | 0.034 ± 0.001^CDfg^ | 0.034 ± 0.001^CDbc^ | 0.032 ± 0.001^EFde^ | 0.02 ± 0.001^Hd^ | ND | 0.033 ± 0.001^DEij^ | 0.028 ± 0.002^Gfg^ | 0.029 ± 0^Ga^ | 0.035 ± 0.001^Ca^ | 0.034 ± 0^CDa^ | ND | ND | ND | ND |
| J2-CF-2h-FP | 0.032 ± 0^Fgh^ | 0.04 ± 0.001^Ba^ | ND | ND | 0.034 ± 0.001^DEfg^ | ND | 0.034 ± 0^DEde^ | 0.021 ± 0.001^Hcd^ | 0.028 ± 0.002^Ga^ | 0.08 ± 0.001^Ad^ | 0.036 ± 0^Ccd^ | ND | 0.035 ± 0^CDa^ | 0.033 ± 0^EFa^ | ND | 0.029 ± 0^Ga^ | ND | 0.036 ± 0^Cde^ |
| J2-CF-2h-CP | 0.032 ± 0^DEgh^ | 0.034 ± 0.001^CDb^ | 0.054 ± 0^Af^ | 0.031 ± 0.001^DEgh^ | 0.042 ± 0.001^Bd^ | 0.032 ± 0^DEc^ | 0.034 ± 0.002^CDde^ | 0.02 ± 0.001^Id^ | 0.026 ± 0.002^GHa^ | 0.041 ± 0^Bfg^ | 0.028 ± 0.001^FGfg^ | 0.029 ± 0.001^EFa^ | 0.036 ± 0.002^Ca^ | ND | 0.024 ± 0^Ha^ | ND | 0.03 ± 0.005^EFa^ | 0.034 ± 0.001^CDfg^ |
| J2-CF-2h-BP | 0.028 ± 0.001^EFk^ | 0.028 ± 0^EFf^ | 0.029 ± 0^Eg^ | 0.036 ± 0.001^Bde^ | 0.032 ± 0.001^Dgh^ | 0.038 ± 0^Ab^ | 0.032 ± 0.001^Dde^ | 0.02 ± 0^Hd^ | 0.026 ± 0^Ga^ | 0.033 ± 0^CDij^ | 0.027 ± 0F^Ggh^ | 0.029 ± 0.001^Ea^ | 0.034 ± 0^Ca^ | 0.034 ± 0.001^Ca^ | ND | 0.028 ± 0^EFa^ | ND | ND |
| J2-CF-4h-FP | 0.033 ± 0^Egh^ | 0.028 ± 0.001^Ff^ | ND | 0.046 ± 0.001^Bab^ | 0.036 ± 0^CDef^ | ND | 0.034 ± 0.001^Ede^ | 0.02 ± 0^Gd^ | 0.029 ± 0.001^Fa^ | 0.089 ± 0.001^Aa^ | 0.038 ± 0.001^Cc^ | ND | 0.035 ± 0^DEa^ | 0.033 ± 0^Ea^ | ND | 0.028 ± 0.005^Fa^ | ND | 0.037 ± 0.002^CDd^ |
| J2-CF-4h-CP | 0.044 ± 0.002^Be^ | 0.028 ± 0^HIf^ | 0.084 ± 0^Ad^ | 0.031 ± 0^EFgh^ | 0.038 ± 0.001^Ce^ | ND | 0.037 ± 0.001^CDc^ | 0.02 ± 0.002^Jd^ | 0.026 ± 0.004^Ia^ | 0.034 ± 0^CDi^ | 0.027 ± 0.005^HIgh^ | 0.029 ± 0.001^GHa^ | 0.035 ± 0.006^CDa^ | ND | ND | ND | 0.03 ± 0.001^FGa^ | 0.033 ± 0.001^DEg^ |
| J2-CF-4h-BP | 0.029 ± 0C^Djk^ | 0.028 ± 0.001^Df^ | ND | 0.036 ± 0^Ade^ | 0.033 ± 0^ABgh^ | 0.034 ± 0.005^ABbc^ | 0.032 ± 0.001^BCde^ | 0.022 ± 0.001^Ebc^ | 0.026 ± 0.002^Da^ | 0.034 ± 0^ABi^ | 0.027 ± 0^Dgh^ | ND | 0.034 ± 0.001^ABa^ | 0.033 ± 0.003^ABa^ | ND | 0.028 ± 0.001^Da^ | ND | ND |
| J2-CF-8h-FP | 0.032 ± 0G^Hgh^ | 0.029 ± 0.002^Ief^ | ND | 0.047 ± 0.001^Bab^ | 0.037 ± 0^Ee^ | ND | 0.031 ± 0.001^Hef^ | 0.02 ± 0^Kd^ | 0.029 ± 0.002^Ia^ | 0.088 ± 0^Aab^ | 0.044 ± 0.001^Cb^ | ND | 0.034 ± 0^Fa^ | 0.033 ± 0^FGa^ | 0.025 ± 0.001^Ja^ | 0.029 ± 0.002^Ia^ | ND | 0.039 ± 0.001^Dc^ |
| J2-CF-8h-CP | 0.046 ± 0^Bde^ | 0.031 ± 0.001^Fde^ | 0.126 ± 0^Ac^ | 0.042 ± 0.004^Cc^ | ND | 0.033 ± 0.001^Fc^ | 0.039 ± 0.001^Dab^ | 0.02 ± 0.001^Hd^ | ND | 0.031 ± 0.001^Fkl^ | 0.027 ± 0.001^Ggh^ | 0.028 ± 0.001^Ga^ | 0.036 ± 0.001^Ea^ | ND | ND | ND | 0.031 ± 0^Fa^ | 0.033 ± 0^Fg^ |
| J2-CF-8h-BP | 0.034 ± 0^Ag^ | ND | ND | 0.033 ± 0.001^Afg^ | 0.031 ± 0.001^ABh^ | ND | 0.031 ± 0^ABef^ | 0.022 ± 0.002^Dbc^ | 0.026 ± 0.001^Ca^ | 0.033 ± 0.001^Aij^ | 0.026 ± 0.001^Ch^ | ND | 0.034 ± 0.002^Aa^ | 0.033 ± 0.005^Aa^ | ND | 0.028 ± 0.001^BCa^ | ND | ND |
| J2-CF-16h-FP | 0.033 ± 0^Dgh^ | ND | ND | 0.047 ± 0.001^Bab^ | 0.036 ± 0.002^Cef^ | ND | 0.031 ± 0^Eef^ | 0.024 ± 0.001^Ga^ | 0.028 ± 0.001^Fa^ | 0.082 ± 0^Ac^ | 0.047 ± 0.001^Bb^ | ND | 0.035 ± 0^Ca^ | 0.033 ± 0.002^Da^ | ND | ND | ND | 0.036 ± 0^Cde^ |
| J2-CF-16h-CP | 0.046 ± 0.001^Cde^ | 0.031 ± 0.002^FGde^ | 0.146 ± 0.001^Aa^ | 0.048 ± 0.001^Ba^ | ND | 0.033 ± 0.001^EFc^ | 0.04 ± 0^Da^ | 0.021 ± 0.001^Icd^ | ND | 0.03 ± 0.001^Gl^ | 0.027 ± 0.001^Hgh^ | ND | 0.034 ± 0.002^Ea^ | ND | ND | ND | 0.031 ± 0^FGa^ | ND |
| J2-CF-16h-BP | 0.033 ± 0^Agh^ | ND | ND | 0.03 ± 0^Bh^ | 0.031 ± 0.001^Bh^ | ND | ND | 0.022 ± 0.001^Ebc^ | 0.026 ± 0^Da^ | 0.033 ± 0.001^Aij^ | 0.027 ± 0^CDgh^ | ND | 0.034 ± 0^Aa^ | 0.033 ± 0.001^Aa^ | ND | 0.028 ± 0.001^Ca^ | ND | ND |
| J2-CF-24h-FP | 0.033 ± 0^Fgh^ | ND | ND | 0.048 ± 0.001^Ca^ | 0.037 ± 0.001^De^ | ND | 0.031 ± 0.001^Gef^ | 0.02 ± 0.001^Id^ | 0.029 ± 0.001^Ha^ | 0.087 ± 0^Ab^ | 0.052 ± 0.001^Ba^ | ND | 0.035 ± 0^Ea^ | 0.033 ± 0.001^Fa^ | ND | 0.029 ± 0^Ha^ | ND | 0.035 ± 0^Eef^ |
| J2-CF-24h-CP | 0.045 ± 0^Bde^ | 0.03 ± 0.001^EFef^ | 0.137 ± 0.002^Ab^ | 0.045 ± 0.001^Bb^ | ND | ND | 0.038 ± 0.003^Cbc^ | 0.02 ± 0^Hd^ | 0.026 ± 0^Ga^ | 0.032 ± 0.001^Ejk^ | 0.028 ± 0.001^FGfg^ | ND | 0.035 ± 0^Da^ | ND | ND | ND | 0.03 ± 0^EFa^ | ND |
| J2-CF-24h-BP | 0.039 ± 0^Af^ | ND | ND | 0.03 ± 0^Ch^ | 0.033 ± 0^Bgh^ | ND | ND | 0.02 ± 0.001^Fd^ | 0.026 ± 0.001^Ea^ | 0.032 ± 0.002^Bjk^ | 0.028 ± 0.001^Dfg^ | ND | 0.034 ± 0.002^Ba^ | 0.033 ± 0^Ba^ | ND | ND | ND | ND |

ND means not detected. The values in each grid represent the mean ± standard deviation. The different uppercase letters in the same row represent significant differences, and the different lowercase letters in the same column represent significant differences ( p < 0.05 ).

Table S4. HPLC quantitative analysis of different forms of polyphenols in jackfruit ( J3 ) during *in vitro* digestion and colon fermentation.

| **Compounds** | **Gallic acid** | **Chlorogenic Acid** | **p-Hydroxybenzoic acid** | **Caffeic acid** | **Homovanillic acid** | **3-Hydroxyphenylacetic acid** | **Sinapic acid** | **Ferulic acid** | **2-Methoxycinnamaldehyde** | **(+)-Cianidanol** | **vitexin-2″-o-rhamnoside** | **Hyperoside** | **(-)-Epicatechin gallate** | **Taxifolin** | **Glycitein** | **Quercetin** | **Naringenin** | **Mulberrin** |
| --- | --- | --- | --- | --- | --- | --- | --- | --- | --- | --- | --- | --- | --- | --- | --- | --- | --- | --- |
| J3-CG-FP | 0.031 ± 0^Bjk^ | ND | 0.029 ± 0.002^CDi^ | ND | 0.034 ± 0.001^Agh^ | ND | ND | ND | ND | 0.031 ± 0^Bk^ | 0.028 ± 0^De^ | ND | ND | ND | ND | 0.030 ± 0.001^BCa^ | ND | ND |
| J3-CG-CP | 0.034 ± 0.001^FGgh^ | 0.031 ± 0.001^HIbc^ | 0.030 ± 0.002^IJi^ | 0.036 ± 0.001^EFb^ | 0.051 ± 0^Cd^ | 0.047 ± 0^Db^ | 0.032 ± 0.001^GHb^ | 0.025 ± 0.001^Ka^ | 0.028 ± 0.002^Jb^ | 0.038 ± 0.002^Ehi^ | 0.068 ± 0.001^Aa^ | 0.030 ± 0^IJb^ | 0.034 ± 0^FGd^ | 0.033 ± 0.002^GHa^ | 0.028 ± 0.001^Ja^ | ND | 0.031 ± 0.001^HIa^ | 0.054 ± 0.001^Ba^ |
| J3-CG-BP | 0.031 ± 0.001^Ejk^ | 0.033 ± 0.002^Db^ | 0.038 ± 0^Cg^ | 0.031 ± 0.001^Eef^ | 0.033 ± 0.001^Dhi^ | 0.060± 0^Aa^ | 0.031 ± 0^Eb^ | 0.025 ± 0^Ga^ | 0.028 ± 0^Fb^ | 0.042 ± 0^Bef^ | 0.042 ± 0.001^Bc^ | ND | 0.033 ± 0^Dd^ | ND | 0.028 ± 0.001^Fa^ | ND | ND | ND |
| J3-GP-FP | 0.043 ± 0.001^Ac^ | 0.028 ± 0^Ed^ | 0.029 ± 0.001^DEi^ | ND | 0.041 ± 0.002^Ae^ | 0.035 ± 0.001^Bd^ | 0.033 ± 0^BCb^ | 0.022 ± 0.001^Fab^ | 0.027 ± 0.002^Eb^ | 0.031 ± 0.001^CDk^ | 0.028 ± 0.002^Ede^ | 0.029 ± 0^DEb^ | ND | ND | ND | ND | ND | 0.033 ± 0^BCd^ |
| J3-GP-CP | 0.06 ± 0.001^Cb^ | 0.031 ± 0.001^HIbc^ | 0.065 ± 0.002^Bc^ | ND | 0.146 ± 0^Aa^ | 0.039 ± 0.001^Fc^ | 0.038 ± 0.001^FGa^ | 0.022 ± 0^Kab^ | 0.027 ± 0.001^Jb^ | 0.043 ± 0.001^Ede^ | 0.028 ± 0.003^Jde^ | 0.032 ± 0.001^Ha^ | 0.036 ± 0.001^Ga^ | 0.032 ± 0.001^Ha^ | ND | 0.029 ± 0.002^IJa^ | ND | 0.048 ± 0.001^Db^ |
| J3-GP-BP | 0.029 ± 0^Bkl^ | ND | 0.029 ± 0^Bi^ | ND | ND | ND | 0.031 ± 0.001^Ab^ | ND | ND | ND | ND | ND | ND | ND | ND | ND | ND | ND |
| J3-IP-FP | 0.031 ± 0.001^ABij^ | ND | ND | ND | ND | ND | 0.032 ± 0^Ab^ | 0.022 ± 0^Dab^ | ND | 0.03 ± 0.001^Bk^ | 0.027 ± 0.001^Ce^ | ND | ND | ND | ND | ND | ND | ND |
| J3-IP-CP | 0.063 ± 0.001^Ba^ | 0.038 ± 0.002^Ea^ | 0.05 ± 0.001^Cd^ | ND | 0.124 ± 0^Ab^ | 0.039 ± 0^Ec^ | 0.038 ± 0^Ea^ | 0.022 ± 0.001^Jab^ | 0.027 ± 0^Hb^ | 0.046 ± 0.00^1Dbc^ | 0.028 ± 0.002^GHde^ | 0.032 ± 0.001^Fa^ | 0.033 ± 0^Fd^ | 0.032 ± 0.001^Fa^ | 0.025 ± 0.002^Ia^ | 0.029 ± 0^Ga^ | ND | 0.047 ± 0^Db^ |
| J3-IP-BP | 0.032 ± 0^Bhi^ | 0.029 ± 0.001^Bcd^ | 0.05 ± 0^Ad^ | 0.031 ± 0^Bde^ | 0.033 ± 0.006^Bgh^ | ND | 0.032 ± 0^Bb^ | 0.023 ± 0.001^Cab^ | ND | 0.031 ± 0.005^Bk^ | ND | ND | ND | ND | ND | ND | ND | 0.033 ± 0^Bd^ |
| J3-CF-0.3h-FP | 0.033 ± 0E^Fgh^ | ND | ND | 0.048 ± 0.001^Ca^ | 0.037 ± 0^Df^ | ND | 0.031 ± 0.004^FGb^ | 0.021 ± 0^Ibc^ | 0.029 ± 0.002^GHa^ | 0.088 ± 0^Aa^ | 0.052 ± 0.001^Bb^ | ND | 0.035 ± 0^DEb^ | 0.033 ± 0.001^EF^ | ND | 0.028 ± 0^Ha^ | ND | 0.036 ± 0^Dc^ |
| J3-CF-0.3h-CP | 0.036 ± 0^De^ | 0.029 ± 0.001^Fcd^ | 0.044 ± 0.002^Be^ | 0.029 ± 0.001^Ff^ | 0.05 ± 0^Ad^ | ND | 0.031 ± 0.001^Eb^ | 0.02 ± 0^Hc^ | 0.026 ± 0^Gb^ | 0.038 ± 0.001^Cgh^ | 0.028 ± 0^Fde^ | ND | ND | ND | ND | ND | ND | 0.036 ± 0.001^Dc^ |
| J3-CF-0.3h-BP | 0.028 ± 0.001^BCl^ | 0.028 ± 0.002^BCd^ | ND | 0.03 ± 0.001^ABef^ | 0.031 ± 0^Ai^ | ND | ND | 0.02 ± 0^Dc^ | ND | 0.03 ± 0.001^ABk^ | 0.027 ± 0.001^Ce^ | ND | ND | ND | ND | ND | ND | ND |
| J3-CF-2h-FP | 0.029 ± 0.001^Ckl^ | ND | ND | 0.031 ± 0.001^Bde^ | ND | ND | 0.031 ± 0^Bb^ | ND | 0.026 ± 0.001^Db^ | 0.04 ± 0^Afg^ | ND | ND | ND | ND | ND | ND | ND | ND |
| J3-CF-2h-CP | 0.035 ± 0.001^CDef^ | 0.032 ± 0.002^DEb^ | 0.041 ± 0.001^Bf^ | 0.03 ± 0.001^EFef^ | 0.05 ± 0^Ad^ | 0.033 ± 0.001^DEd^ | 0.031 ± 0.006^DEb^ | 0.02 ± 0.003^Hc^ | 0.026 ± 0.001^Gb^ | 0.038 ± 0^BCgh^ | 0.028 ± 0.001^FGde^ | ND | ND | ND | ND | ND | ND | ND |
| J3-CF-2h-BP | 0.028 ± 0.002^Cl^ | ND | ND | 0.031 ± 0^Bde^ | 0.034 ± 0^Agh^ | ND | 0.031 ± 0.001^Bb^ | 0.02 ± 0^Ec^ | 0.026 ± 0.001^Db^ | 0.031 ± 0.001^Bk^ | 0.028 ± 0^Cde^ | ND | ND | ND | ND | ND | ND | ND |
| J3-CF-4h-FP | 0.03 ± 0.001^Cjk^ | ND | ND | 0.033 ± 0^Bc^ | ND | ND | 0.031 ± 0.001^Cb^ | ND | 0.026 ± 0^Eb^ | 0.043 ± 0^Ade^ | 0.028 ± 0.001^Dde^ | ND | ND | ND | ND | ND | ND | ND |
| J3-CF-4h-CP | 0.039 ± 0^Cd^ | 0.039 ± 0.001^Ca^ | 0.063 ± 0.002^Ac^ | 0.03 ± 0.001^Fef^ | 0.053 ± 0.002^Bc^ | 0.034 ± 0.003^DEd^ | 0.032 ± 0.001^EFb^ | 0.02 ± 0^Hc^ | ND | 0.035 ± 0^Dij^ | 0.027 ± 0^Ge^ | ND | ND | ND | ND | ND | ND | ND |
| J3-CF-4h-BP | 0.028 ± 0.001^Cl^ | 0.028 ± 0.002^Cd^ | ND | 0.03 ± 0.001^Bef^ | 0.042 ± 0^Ae^ | ND | ND | 0.02 ± 0^Ec^ | 0.026 ± 0^Db^ | 0.031 ± 0.001^Bk^ | 0.028 ± 0^Cde^ | ND | ND | ND | ND | ND | ND | ND |
| J3-CF-8h-FP | 0.029 ± 0^Ckl^ | ND | ND | 0.036 ± 0.001^Bb^ | ND | ND | 0.031 ± 0.003^Cb^ | 0.02 ± 0.001^Ec^ | 0.026 ± 0.001^Db^ | 0.045 ± 0^Acd^ | 0.03 ± 0^Cd^ | ND | ND | ND | ND | ND | ND | ND |
| J3-CF-8h-CP | 0.042 ± 0.001^Bc^ | 0.032 ± 0.002^Cb^ | 0.086 ± 0^Aa^ | 0.03 ± 0.001^Cef^ | 0.041 ± 0^Be^ | ND | ND | ND | ND | 0.03 ± 0.001^Ck^ | 0.027 ± 0.002^De^ | ND | ND | ND | ND | ND | ND | ND |
| J3-CF-8h-BP | 0.028 ± 0.001^CDl^ | 0.031 ± 0.002^ABbc^ | ND | 0.031 ± 0.001^ABde^ | 0.032 ± 0.001^Ahi^ | ND | ND | ND | 0.026 ± 0.001^Db^ | 0.029 ± 0.001^BCk^ | 0.03 ± 0.002^ABd^ | ND | ND | ND | ND | ND | ND | ND |
| J3-CF-16h-FP | 0.03 ± 0.001^Cjk^ | ND | ND | 0.033 ± 0^Bc^ | ND | ND | 0.031 ± 0^Cb^ | 0.02 ± 0.001^Ec^ | 0.026 ± 0^Db^ | 0.048 ± 0^Ab^ | ND | ND | ND | ND | ND | ND | ND | ND |
| J3-CF-16h-CP | 0.034 ± 0.001^BCfg^ | 0.03 ± 0.001^Dbc^ | 0.083 ± 0^Ab^ | 0.032 ± 0^CDcd^ | 0.035 ± 0.001^Bfg^ | ND | 0.031 ± 0^Db^ | ND | ND | 0.03 ± 0.001^Dk^ | 0.027 ± 0.003^Ee^ | ND | ND | ND | ND | ND | ND | ND |
| J3-CF-16h-BP | 0.029 ± 0.001^CDkl^ | 0.03 ± 0.001^Cbc^ | 0.029 ± 0.002^CDi^ | ND | 0.035 ± 0^Bfg^ | 0.039 ± 0.001^Ac^ | ND | 0.02 ± 0.002^Fc^ | 0.026 ± 0^Eb^ | 0.034 ± 0.001^Bj^ | 0.027 ± 0.002^DEe^ | ND | 0.034 ± 0^Bc^ | ND | ND | ND | ND | ND |
| J3-CF-24h-FP | 0.03 ± 0^Djk^ | ND | ND | 0.033 ± 0.001^Bc^ | 0.032 ± 0.001^BChi^ | ND | 0.031 ± 0^CDb^ | 0.02 ± 0^Fc^ | 0.026 ± 0^Eb^ | 0.047 ± 0.001^Abc^ | ND | ND | ND | ND | ND | ND | ND | ND |
| J3-CF-24h-CP | 0.033 ± 0^CDgh^ | 0.03 ± 0^Ebc^ | 0.084 ± 0.001^Aab^ | ND | 0.04 ± 0^Be^ | ND | 0.031 ± 0.001^DEb^ | 0.02 ± 0.003^Gc^ | ND | 0.031 ± 0.001^DEk^ | 0.026 ± 0.001^Fe^ | ND | 0.034 ± 0^Cc^ | ND | ND | ND | ND | ND |
| J3-CF-24h-BP | 0.031 ± 0^BCij^ | 0.028 ± 0^DEd^ | 0.032 ± 0.001^Bh^ | 0.029 ± 0.002^CDf^ | ND | 0.044 ± 0.001^Ab^ | 0.031 ± 0.003^BCb^ | ND | 0.026 ± 0.001^Eb^ | ND | 0.027 ± 0.001^DEe^ | 0.028 ± 0.002^DEa^ | ND | ND | ND | 0.028 ± 0.002^DEa^ | ND | 0.032 ± 0.001^Bd^ |

ND means not detected. The values in each grid represent the mean ± standard deviation. The different uppercase letters in the same row represent significant differences, and the different lowercase letters in the same column represent significant differences ( p < 0.05 ).

Table S5. Analysis of α-diversity of intestinal microbial community during *in vitro* colonic fermentation of jackfruit with different maturity.

| **Group** | **observed species** | | | **Shannon** | | | **Simpson** | | | **Chao1** | | | **ACE** | | |
| --- | --- | --- | --- | --- | --- | --- | --- | --- | --- | --- | --- | --- | --- | --- | --- |
|  | **J1** | **J2** | **J3** | **J1** | **J2** | **J3** | **J1** | **J2** | **J3** | **J1** | **J2** | **J3** | **J1** | **J2** | **J3** |
| CF-0.3h | 136.33 ± 2.89^Aa^ | 138 ± 2^Aa^ | 134 ± 3.61^Aa^ | 3.24 ± 0.04^Aa^ | 3.32 ± 0.2^Aa^ | 3.17 ± 0.06^Aa^ | 0.93 ± 0^Ba^ | 0.93 ± 0.01^Aa^ | 0.92 ± 0.01^Aa^ | 136.51 ± 2.92^Aa^ | 139.23 ± 1.75^Aa^ | 134.56 ± 3.95^Aa^ | 136.84 ± 2.85^Aa^ | 139.08 ± 2.02^Aa^ | 134.64 ± 3.8^Aa^ |
| CF-2h | 136 ± 2.65^Ba^ | 131.67 ± 3.21^ABa^ | 132.33 ± 4.04^Aa^ | 3.34 ± 0.19^Aa^ | 3.15 ± 0.13A^Ba^ | 3.17 ± 0.11^Aa^ | 0.93 ± 0.01^Aa^ | 0.92 ± 0.01^Aa^ | 0.92 ± 0.01^Aa^ | 137.73 ± 4.41^Aa^ | 132.82 ± 1.4^ABa^ | 133.67 ± 3.21^Aa^ | 136.76 ± 2.84^Ba^ | 132.43 ± 2.63^ABa^ | 133.43 ± 3.27^ABa^ |
| CF-4h | 125.67 ± 8.02^Ca^ | 120.67 ± 5.51^Ba^ | 117.33 ± 26.5^ABa^ | 2.5 ± 0.02^Bb^ | 2.24 ± 0.06^CDc^ | 2.8 ± 0.15^Ba^ | 0.82 ± 0.02^Cab^ | 0.77 ± 0.04^Bb^ | 0.88 ± 0.04^ABa^ | 129.8 ± 10.38^Aa^ | 126.44 ± 5.53^Ba^ | 119.56 ± 26.21^ABa^ | 128.57 ± 8.94^CDa^ | 125.76 ± 4.43^Ba^ | 119.18 ± 26.05^ABa^ |
| CF-8h | 113.67 ± 3.51^Dab^ | 106.67 ± 14.57^Cb^ | 127.33 ± 1.53^ABa^ | 2.01 ± 0.06^Da^ | 1.86 ± 0.46^Da^ | 1.83 ± 0.08^Ca^ | 0.69 ± 0.02^Da^ | 0.63 ± 0.16^Ca^ | 0.58 ± 0.02^Da^ | 119.19 ± 3.75^Bab^ | 108.45 ± 14.32^Cb^ | 130.86 ± 2.6^ABa^ | 119.82 ± 3.4^DEab^ | 109.51 ± 13.71^Cb^ | 130.95 ± 1.42^ABa^ |
| CF-16h | 110.67 ± 5.69^Da^ | 96.33 ± 10.6^Ca^ | 109.33 ± 3.06B^Ca^ | 2 ± 0.19^Da^ | 2.19 ± 0.17^CDa^ | 1.61 ± 0.17^CDb^ | 0.69 ± 0.05^Dab^ | 0.8 ± 0.02^Ba^ | 0.63 ± 0.09^Db^ | 115.51 ± 3.36^Ba^ | 99.49 ± 6.92^Cb^ | 112.37 ± 3.89B^Ca^ | 115.42 ± 4.97^Ea^ | 100.67 ± 7.1^Cb^ | 113.86 ± 3.92^BCa^ |
| CF-24h | 113.67 ± 5.51^Da^ | 104.67 ± 1.15^Ca^ | 91.33 ± 7.02^Cb^ | 2.22 ± 0.02^Cb^ | 2.36 ± 0.05^Ca^ | 1.68 ± 0.04^Dc^ | 0.8 ± 0^Cb^ | 0.84 ± 0.02^ABa^ | 0.71 ± 0.03^Cc^ | 117.12 ± 5.89^Ba^ | 105.55 ± 1.96^Cb^ | 97.45 ± 2.5^Cc^ | 117.86 ± 5.83^Ea^ | 106.35 ± 2.61^Cb^ | 98.19 ± 6.43^Db^ |
| Control | 133 ± 2.65 | | | 3.67 ± 0.1 | | | 0.95 ± 0.01 | | | 134.92 ± 4.27 | | | 134.26 ± 3.48 | | |

The value in each grid represents the mean ± standard deviation. Different capital letters represent the same maturity and the same α-diversity index, and there are significant differences in different digestion stages. Different lowercase letters represent the same digestion stage and the same α-diversity index, and there are significant differences between different maturity ( p < 0.05 ).

Table S6. Determination of SCFAs content in different maturity jackfruit during *in vitro* colonic fermentation. Abbreviation : Control, fresh slurry control.

| **Group** | **Butyric acid** | | | **Isobutyric acid** | | | **Isovaleric acid** | | | **Propionic acid** | | | **Valeric acid** | | | **Acetic acid** | | | **Total SCFAs** | | |
| --- | --- | --- | --- | --- | --- | --- | --- | --- | --- | --- | --- | --- | --- | --- | --- | --- | --- | --- | --- | --- | --- |
|  | **J1** | **J2** | **J3** | **J1** | **J2** | **J3** | **J1** | **J2** | **J3** | **J1** | **J2** | **J3** | **J1** | **J2** | **J3** | **J1** | **J2** | **J3** | **J1** | **J2** | **J3** |
| CF-0.3h | 0.31 ± 0.01^Da^ | 0.31 ± 0.01^Ca^ | 0.15 ± 0.01^Cb^ | 0.02 ± 0^Ca^ | 0.02 ± 0^Ca^ | 0.01 ± 0^Db^ | 0.02 ± 0^Da^ | 0.02 ± 0^Ca^ | 0.01 ± 0^Cb^ | 1.46 ± 0.01^Db^ | 1.54 ± 0.03^Ca^ | 0.56 ± 0.01^Cc^ | 0.08 ± 0^Ba^ | 0.08 ± 0^Ca^ | 0.03 ± 0^Cb^ | 3.15 ± 0.24^Fa^ | 3.24 ± 0.08^Da^ | 1.43 ± 0.1^Fb^ | 5.04 ± 0.27^Ea^ | 5.22 ± 0.12^Da^ | 2.2 ± 0.1^Fb^ |
| CF-2h | 0.19 ± 0.01^Ea^ | 0.14 ± 0.01^Cb^ | 0.08 ± 0.01^Cc^ | 0.02 ± 0^Ca^ | 0.02 ± 0^Ca^ | 0.01 ± 0^Db^ | 0.02 ± 0^Da^ | 0.02 ± 0^Ca^ | 0.01 ± 0^Cb^ | 1.45 ± 0.07^Da^ | 1.49 ± 0^Ca^ | 0.55 ± 0.04^Cb^ | 0.05 ± 0^CDa^ | 0.04 ± 0^Cb^ | 0.02 ± 0^Cc^ | 5.18 ± 0.22^Eb^ | 5.87 ± 0.33^Da^ | 2.25 ± 0.16^Ec^ | 6.92 ± 0.29^Eb^ | 7.59 ± 0.34^Da^ | 2.91 ± 0.21^Ec^ |
| CF-4h | 0.09 ± 0^Fa^ | 0.08 ± 0^Cb^ | 0.05 ± 0^Cc^ | 0.02 ± 0^Ca^ | 0.02 ± 0^Ca^ | 0.01 ± 0^Db^ | 0.02 ± 0^Da^ | 0.02 ± 0^Ca^ | 0.01 ± 0^Cb^ | 1.4 ± 0.06^Da^ | 1.32 ± 0.01^Cb^ | 0.58 ± 0.01^Cc^ | 0.03 ± 0^Da^ | 0.03 ± 0^Cb^ | 0.01 ± 0^Cc^ | 10.57 ± 0.46^Da^ | 10.49 ± 0.15^Da^ | 3.01 ± 0.08^Db^ | 12.13 ± 0.52^Da^ | 11.96 ± 0.16^Da^ | 3.67 ± 0.1^Db^ |
| CF-8h | 0.66 ± 0.01^Ca^ | 0.65 ± 0.02^Ca^ | 0.18 ± 0^Cb^ | 0.03 ± 0^Ba^ | 0.03 ± 0^BCa^ | 0.01 ± 0^Cb^ | 0.03 ± 0^Ca^ | 0.03 ± 0^Ca^ | 0.01 ± 0^Cb^ | 2.56 ± 0.04^Ca^ | 2.26 ± 0.08^Cb^ | 0.57 ± 0.01^Cc^ | 0.05 ± 0C^Da^ | 0.05 ± 0^Ca^ | 0.02 ± 0^Cb^ | 31.46 ± 0.18^Ca^ | 29.09 ± 1^Cb^ | 12.91 ± 0.72^Cc^ | 34.78 ± 0.22^Ca^ | 32.11 ± 1.1^Cb^ | 13.71 ± 0.74^Cc^ |
| CF-16h | 1.11 ± 0.01^Bc^ | 6.08 ± 0.04^Ba^ | 2.43 ± 0.01^Bb^ | 0.03 ± 0^Bb^ | 0.04 ± 0^Ba^ | 0.02 ± 0^Bc^ | 0.03 ± 0^Bc^ | 0.05 ± 0^Ba^ | 0.03 ± 0^Bb^ | 9.17 ± 0.4^Bb^ | 15.08 ± 0.19^Ba^ | 4.25 ± 0.16^Bc^ | 0.06 ± 0^BCc^ | 0.48 ± 0.01^Ba^ | 0.42 ± 0.01^Bb^ | 43.17 ± 1.83^Bb^ | 79.36 ± 0.79^Ba^ | 19.26 ± 0.15^Bc^ | 53.57 ± 2.21^Bb^ | 101.09 ± 0.64^Ba^ | 26.42 ± 0.24^Bc^ |
| CF-24h | 14.72 ± 0.14^Ab^ | 17.43 ± 1.4^Aa^ | 10.3 ± 0.31^Ac^ | 0.11 ± 0^Ab^ | 0.09 ± 0.01^Ac^ | 0.15 ± 0^Aa^ | 0.24 ± 0^Ab^ | 0.18 ± 0.02^Ac^ | 0.36 ± 0.01^Aa^ | 17.27 ± 0.21^Ab^ | 19.61 ± 1.76^Aa^ | 6.25 ± 0.07^Ac^ | 3.51 ± 0.04^Aa^ | 3.89 ± 0.45^Aa^ | 3.7 ± 0.14^Aa^ | 74.05 ± 1.85^Ab^ | 89.97 ± 11.61^Aa^ | 24 ± 0.08^Ac^ | 109.89 ± 2.18^Ab^ | 131.17 ± 15.25^Aa^ | 44.76 ± 0.5^Ac^ |
| Control | 0.01 ± 0 | | | 0 ± 0 | | | 0 ± 0 | | | 0.03 ± 0 | | | 0 ± 0 | | | 0.06 ± 0 | | | 0.11 ± 0.01 | | |

The value in each grid represents the mean ± standard deviation. Different capital letters represent the same maturity and the same fatty acid, and there are significant differences in different digestion stages. Different lowercase letters represent the same digestion stage, the same fatty acid, and there are significant differences between different maturity ( p < 0.05 ).
